# Facile synthesis of Zn-Mn bimetallic MOF for adsorptive removal of methyl paraben from aqueous solution

**DOI:** 10.1007/s11356-026-37959-7

**Published:** 2026-07-02

**Authors:** Anusha D. Shetty, Athira Balachandran Nair, Harshini Dasari, Nethaji Sundarabal

**Affiliations:** Manipal Institute of Technology, Manipal Academy of Higher Education, Manipal, India

**Keywords:** Adsorption, Bimetallic metal-organic frameworks, Emerging pollutant, Water treatment

## Abstract

A zinc-manganese bimetallic metal-organic framework (Zn-Mn MOF) was synthesised using room temperature, and its adsorption performance was evaluated against methyl paraben (MePr), an emerging endocrine-disrupting compound. The adsorbent exhibited high removal efficiencies for MePr, corresponding to the synergistic effect of Zn and Mn, and its high specific surface area (SSA) of 1657.2 m^2^/g. The synthesised MOF was characterised using FTIR, Raman spectroscopy, XRD, FE-SEM, and BET analyses, which revealed the development of a microporous framework with a hexagonal morphology and high SSA. FTIR verified incorporation of the 2-methylimidazole linker, while XRD showed sharp ZIF-8-type reflections with slight shifts attributable to Mn incorporation; XPS further confirmed the chemical states of the metal centres within the framework. To conduct adsorption batch studies, pH, adsorbent dosage, concentration, contact time, and temperature were chosen as variables. The kinetic analysis estimated that the adsorption followed pseudo-second order kinetics with *R*^2^ > 0.99. The adsorption isotherm studies were defined by the Langmuir model, achieving a maximum adsorption capacity of 52.38 mg/g. The adsorption thermodynamics confirmed that the process is spontaneous and exothermic. Key mechanisms include hydrogen bonding with N/O sites, π-π stacking through imidazole linkers, and electrostatic interactions from Zn-Mn centres. The removal performance of methyl paraben was evaluated by spiking it into various real-world water matrices under diverse water quality conditions. Reusability studies further confirmed the effective performance of the prepared adsorbent in multiple cycles of operation. The results established that Zn-Mn MOF can be an efficient adsorbent for emerging organic contaminants.

## Introduction

Pollution from organic contaminants in wastewater is escalating due to the increasing use of pharmaceuticals and personal care products (PCPs) (Akhtar et al. 2021). Rising concentrations of organic pollutants in aquatic environments pose dual risks to both ecosystems and human health by inducing oxidative stress and toxicity in aquatic organisms while contaminating drinking water supplies (Diogo et al. 2023; Vieira et al. 2022).

Among emerging organic contaminants, parabens are a group of synthetic alkyl esters of p-hydroxybenzoic acid, conventionally used as preservatives in pharmaceuticals and PCPs (Sousa et al. 2023). Parabens commonly detected in aqueous systems include methyl paraben, propylparaben, ethylparaben, and butylparaben. Among these, studies have consistently shown that MePr is predominant in both aqueous phases and sludge matrices, as it is frequently used as an antibacterial in health care and cosmetic products (Li et al. 2015).

MePr have been linked to endocrine disruption in humans and may exhibit carcinogenic effects when interacting with chlorine in wastewater. Additionally, studies indicate that persistent exposure to MePr is linked with an increased incidence of obesity, infertility, and hypersensitivity. They have also been known to cause developmental disorders in aquatic life (Chatterjee et al. 2024; Janiga-macnelly et al. 2025).

A variety of separation processes are used for the elimination of MePr, including adsorption, biodegradation, membrane filtration, and photocatalytic degradation. However, most of these advanced technologies are highly energy-intensive and involve complex operational procedures (Ramutshatsha-Makhwedzha and Munonde 2024; Zhang et al. 2024).

Among these methods, adsorption has remained the preferred separation technique because of its economic viability, efficiency, operational simplicity, and ability to operate under optimised conditions. Adsorption is a phenomenon that relies on the interaction between contaminants and adsorbent surfaces, where removal efficiency is directly driven by the adsorbent’s pore structure, surface area, and chemical affinity for target pollutants. However, the selection of an appropriate adsorbent is a key factor since it profoundly affects the adsorption capacity (Kumar et al. 2024).

MOFs (metal-organic frameworks) are extensively used for their superior adsorption properties (Ahmad et al. 2020). MOFs comprise one or more metal ions and organic linkers. Some of their characteristics that make them useful as adsorbents include high surface area, porosity, and surface functionality (Alsehli 2023; Essalmi et al. 2024). These materials demonstrate exceptional selectivity in adsorption, enabled by the facile customisation of metal ions, organic linkers, and solvents. Their efficacy stems particularly from engineered surface functional groups and precisely tuned pore characteristics, which enhance contaminant binding and uptake efficiency (Abid et al. 2024).

Belonging to the zeolitic class of MOFs, the ZIF-8 MOF has been used as an adsorbent for various pollutants (Ahmad et al. 2021). This class of MOFs consists of the M-Im-M structure, where the M represents Zn^+^ and the Im refers to the imidazolates. The zeolitic framework combines the advantages of conventional MOFs with the stability provided by the zeolitic skeletons, similar to aluminosilicate-based zeolites. However, certain limitations have been associated with the performance of the ZIF-8 MOF due to limited functional groups. Hence, it has been beneficial to combine different metal ions with the ZIF-8 MOF for its high performance and stability (Ahmad et al. 2022; Altaf et al. 2024a).

Bimetallic metal-organic frameworks, incorporating two distinct metal ions, have gathered significant attention due to their enhanced properties compared to monometallic counterparts. It has been shown to have a higher surface area and a favourable active site for adsorption (Luo et al. 2023; Yang and Bai 2019). Bimetallic MOFs demonstrate markedly enhanced adsorption performance over their single-metal counterparts due to optimised structural modifications. For example, Nazir et al. synthesised Mn@ZIF-8 through the solvothermal method, where the adsorption of methyl orange resulted in an SSA of 1257 m^2^/g, achieving a maximum adsorption capacity (*q*_max_) of 406 mg/g, greater than pristine ZIF-8 (307 mg/g) (Altaf et al. 2024b).

While bimetallic MOFs combining Zn with Cu, Co, Fe, and Ni have been extensively explored for adsorption, Zn-Mn variants remain underexplored, particularly for targeted pollutant removal. For example, a copper-zinc benzene tricarboxylic acid MOF (Cu-Zn-BTC MOF) was synthesised, which exhibits a *q*_max_ of 339.5 mg/g, substantially higher than monometallic Cu-BTC MOF (Momin et al. 2024).

In the present study, ZIF-8, Mn-MOF, and Zn-Mn bimetallic MOF were synthesised using 2-methylimidazole as the organic linker. The adopted approach represents a green and energy-efficient alternative to conventional hydrothermal/solvothermal methods, resulting in materials with exceptionally high surface area.

Zn and Mn were selected for bimetallic MOF formation due to their complementary properties that increase the adsorption performance of the MePr. Zn^2+^ provides material with high surface area and framework stability characteristic of ZIF-8, while Mn^2+^ introduces Lewis’s acidity, variable coordination geometries, and Jahn-Teller distortion effects that create additional active sites for generating additional active sites that promote strong interactions with the aromatic and functional groups of MePr. The difference in the ionic radii of Zn^2+^ (74 pm) and Mn^2+^ (83 pm) and the electronic configurations of Zn ([Ar]3d^10^) and Mn ([Ar]3d^5^) enable lattice strain and synergistic metal-ligand interactions, promoting π-π stacking, hydrogen bonding, and electrostatic attraction with methyl paraben’s polar or aromatic functionalities.

This study represents one of the most comprehensive investigations of the adsorption performance of the Zn-Mn bimetallic system to date. The material was thoroughly characterised using XRD, XPS, BET, FE-SEM, FTIR, and Raman spectroscopy, demonstrating robust multi-cycle reusability and strong efficacy in real water matrices. Post-adsorption characterisation confirmed good structural retention of the MOF, further supporting its stability during pollutant uptake. These findings establish scalable structure-property relationships with significant implications for wastewater remediation applications.

## Materials and methods

### Chemicals and reagents

Zinc nitrate hexahydrate (Zn (NO_3_)_2_∙6H_2_O, 96%), manganese (II) nitrate tetrahydrate (Mn (NO_3_)_2_·4H_2_O, 97%), and 2-methylimidazole (C_4_H_6_N_2_, 99%) were procured from Loba Chemicals, India. Absolute ethanol (C_2_H_5_OH, 99%) was procured from Changshu Hongheng Fine Chemical Co. Ltd., and methanol (CH_3_OH, Extrapure) was purchased from Actylis. Methyl paraben (C_8_H_8_O_3,_ Extra Pure) was obtained from Sigma-Aldrich. All the reagents were used without further purification.

### Synthesis of monometallic MOFs, ZIF-8, and Mn-MOF

A one-pot room-temperature method was used to synthesise ZIF-8. Specifically, 8.49 mmol 2-methylimidazole was dissolved in methanol, while 1.99 mmol Zn (NO₃)₂·6H₂O was dissolved separately in CH_3_OH. The solutions were stirred at room temperature, yielding a white precipitate that was separated, washed with methanol and dried overnight at 80 °C (Tahir et al. 2020).

Mn-MOF was synthesised through a precipitation method. For this, 1.89 mmol Mn (NO₃)₂·4H₂O was dissolved in methanol, while 8.49 mmol 2-methylimidazole (Hmim) was separately dissolved in methanol. The solutions were mixed and stirred, transferred to a closed container, and aged for 48 h. The pale brown precipitate formed was washed with methanol and dried at 60 °C (Shamshul et al. 2024).

### Synthesis of bimetallic MOFs, Zn-Mn MOF

Zn-Mn MOF was synthesised through a facile room-temperature process using a fixed Zn:Mn molar ratio of 75:25 selected based on screening multiple ratios that demonstrated optimal MePr removal efficiency. Specifically, 3.75 mmol Zn(NO₃)₂·6H₂O and 1.25 mmol Mn (NO₃)₂·4H₂O were dissolved in methanol, followed by the addition of 2-methylimidazole (Hmim:total metal = 8:1). The mixture was stirred overnight to promote homogeneous nucleation and bimetallic incorporation. The precipitate was then separated and collected by centrifugation, washing thrice with methanol and drying at 80 °C overnight to yield a pale-yellow powder.

### Characterisation techniques

The prepared MOFs were characterised using BET, FTIR, FE-SEM, EDS, XRD, Raman, and XPS. Brunauer-Emmett-Teller (BET) surface analyser, MICROTRAC-BELSORP MINI X, was employed for computing the surface area and the pore volume of MOFs. Fourier transform infrared spectroscopy (FTIR) (Bruker Alpha II) was used to detect the functional groups. The surface and morphological features were observed using field emission scanning electron microscopy (FE-SEM), coupled with energy dispersive spectroscopy (EDS) for elemental mapping and confirmation (GEMINI 300, Carl Zeiss, Germany). X-ray diffractometry (XRD) was used to characterise the adsorbent’s crystallographic structure (Empyrean 3rd Gen, Malvern PANalytical, Netherlands). Additionally, the structural fingerprint of the MOF was identified using Raman spectroscopy (Wasatch Photonics, 830 nm laser). X-ray photoelectron spectroscopy (XPS) (K-ALPHA) was used to analyse surface chemical composition and elemental states of the synthesised material.

### Batch adsorption studies

A batch adsorption study was carried out by varying the parameters such as aqueous pH of the solution, initial adsorbate concentration, adsorbent dosage, temperature, and contact time. A stock solution of MePr (1000 mg/L) was prepared, from which working solutions were prepared by dilution with water as required for each experimental parameter. The aqueous pH was adjusted with 0.1 N NaOH and 0.1 N HCl solutions. Following the addition of the optimal adsorbent dosage to a 10 mg/L pollutant solution, standard adsorption experiments were conducted in an incubator shaker. The adsorbate was separated from the solution, and the concentration of paraben was obtained using UV spectroscopy.

The removal efficiency and adsorption capacity (*q*_e_) were calculated using Eqs. (1) and (2) (Mohan et al. 2025).

1 $$\eta \left(\mathrm{\%}\right)=\frac{{c}_{0}-{c}_{e}}{{c}_{0}}\times 100$$

where *C*_o_ and *C*_e_ are the initial concentration and the concentration at equilibrium.

2 $${q}_{\mathrm{e}}=\frac{\left({c}_{0}-{c}_{\mathrm{e}}\right)V}{W}$$

${q}_{\mathrm{e}}$ represents adsorption capacity at equilibrium, and ${c}_{0} \mathrm{a}\mathrm{n}\mathrm{d} {c}_{\mathrm{e}}$ are the adsorbate concentrations at the initial time and at equilibrium. $W$ is the mass of the adsorbent, and $V$ is the volume of the aqueous phase.

### Adsorption isotherm

The adsorption characteristics of parabens onto the Zn-Mn MOF were modelled using the Freundlich, Langmuir, and Temkin isotherms.

The Langmuir isotherm is given by Eq. 3 (Wang and Guo 2020b).

3 $${q}_{\mathrm{e}}=\frac{{q}_{m}{K}_{\mathrm{L}}{C}_{\mathrm{e}}}{1+{K}_{\mathrm{L}}{C}_{\mathrm{e}}}$$

where ${K}_{\mathrm{L}}$ is the Langmuir constant, and ${C}_{\mathrm{e}}$ is the equilibrium concentration of the adsorbate.

The Freundlich isotherm is given by Eq. 4 (Zhou et al. 2018).

4 $${q}_{\mathrm{e}}={K}_{\mathrm{F}}{C}_{e}^\frac{1}{n}$$

where ${K}_{\mathrm{F}}$ is the distribution coefficient, and *n* is the correction factor.

The Temkin isotherm is given by Eq. 5 (Dziejarski 2023).

5 $${q}_{\mathrm{e}}=\frac{RT}{{b}_{\mathrm{T}}}\mathrm{ln}\left({A}_{\mathrm{T}}{C}_{\mathrm{e}}\right)$$

where *R* is the universal gas constant, *T* is the temperature, and *b*_T_ is the adsorption energy.

### Adsorption kinetics

The kinetic behaviour of the adsorption process was evaluated using pseudo-first-order (PFO) and pseudo-second-order (PSO) kinetic models.

The PFO kinetic model is given by Eq. 6 (Wang and Guo 2020a).

6 $${q}_{\mathrm{t}}={q}_{\mathrm{e}}({1-e}^{-k_1t})$$

where ${k}_{1}$ is the rate constant for the PFO model.

The PSO kinetic model is given by Eq. 7 (Essam et al. 2024).

7 $$q_t = \frac{k_2 q_e^2 \, t}{1 + k_2 q_e \, t}$$

where ${k}_{2}$ is the rate constant for PSO models.

The intraparticle diffusion (IPD) model is given by Eq. 8 (Wu et al. 2009).

8 $${{q}_{\mathrm{t}}=K}_{\mathrm{i}\mathrm{d}}{t}^\frac{1}{2}+C$$

where ${K}_{\mathrm{i}\mathrm{d}}$ represents the intraparticle diffusion rate constant values, and *C* is the intercept.

The Elovich model is given by Eq. 9 (Acharya et al. 2024).

9 $${q}_{\mathrm{t}}=\left(\frac{1}{\beta }\right)\mathrm{l}\mathrm{n}(1+\alpha \beta t)$$

where $\alpha$ is the initial adsorption rate, and $\beta$ is the desorption constant.

### Adsorption thermodynamics

Thermodynamic parameters were determined to assess the feasibility of the MePr adsorption onto Zn-Mn MOF by calculating the Gibbs free energy$(\Delta {G}^{\circ })$, standard enthalpy $(\Delta {H}^{\circ })$, and standard entropy $\left(\Delta {S}^{\circ }\right)$ at different temperatures based on the Langmuir equilibrium constant ${K}_{\mathrm{L}}$. Adsorption thermodynamic parameters are calculated using Eqs. 10 and 11 (Atheba et al. 2018).

10 $$\Delta {G}^{\circ }=\Delta {H}^{\circ }-T\Delta S^\circ$$

11 $$\Delta {G}^{\circ }= -RT\mathrm{ln}{K}_{L}$$

Where ${K}_{L}$ is the Langmuir constant, $R$ is the universal gas constant, and $T$ signifies the absolute temperature.

### Reusability study of Zn-Mn MOF

The reusability of the Zn-Mn MOF adsorbent for the removal of MePr was tested through cyclic adsorption and desorption experiments under optimised conditions. After each adsorption experiment, the used MOF was filtered, washed with distilled water, and dried. Additionally, the MOF was treated with ethanol as the solvent for desorption. The choice of ethanol as the desorption solvent was based on its ability to overcome hydrophobic and π-π interactions between MePr and the MOF. Moreover, ethanol has been widely recognised in the literature as a non-toxic, cost-effective, and efficient regenerative solvent for paraben removal. In contrast to other chemical agents, such as acids and strong bases, ethanol is relatively mild, preventing the collapse of the MOF framework (Bernal et al. 2019; Ran et al. 2020). The MOF was then dried at 80 °C between cycles to confirm removal of solvent and restoration of adsorption sites. This procedure was repeated for three cycles, and the *q*_e_ was measured after each regeneration to assess the economic and operational viability of the MOF.

### Matrix spiking study

Spiking experiments were performed to assess the efficiency of Zn-Mn MOF in MePr removal at different water samples. The real water samples of the lake, river, tap, and seawater were collected from the Udupi region using sterilised containers. The water samples were filtered to remove particles to minimise external interferences. MePr was spiked into the water samples at mg/L levels, which are the optimum levels for adsorption. The MePr levels present in the water samples of the region are at trace levels of ng/L. However, spiking was done at mg/L levels due to quantification requirements and material constraints (Li et al. 2015).

## Results and discussion

### Characterisation of the Zn-Mn MOF

**Fig. 1:**
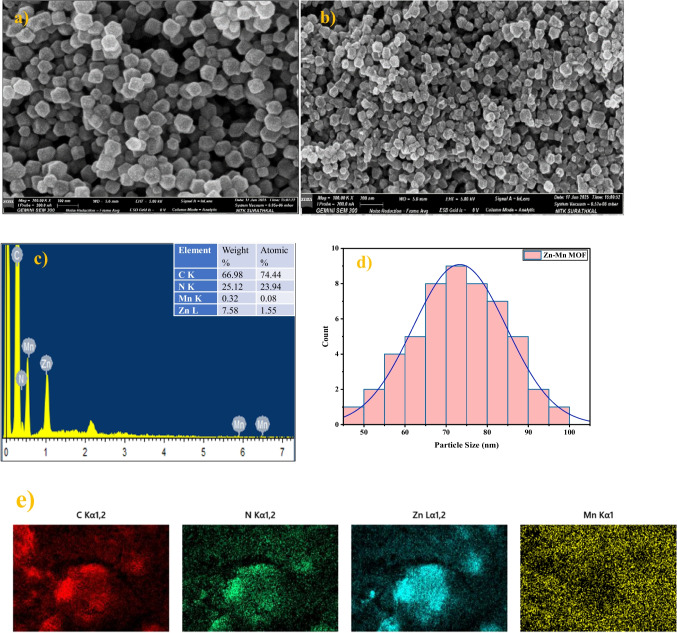
**a**, **b** FE-SEM images, **c** EDS, **d** histogram, and **e** elemental mapping images of Zn-Mn MOF

FE-SEM images of Zn-Mn MOF are represented in Fig. 1a and b. The particles were observed to be polyhedral particles with rhombic dodecahedral-like morphology, which was typical of ZIF-8-derived metal-organic frameworks. This indicates that the Zn-Mn MOF was formed through a controlled synthesis procedure, yielding discrete nano- to micro-sized crystallites (Wan et al. 2021). Elemental confirmation in Zn-Mn MOF was determined using EDS, as depicted in Fig. 1c. EDS analysis confirmed the presence of C, N, Zn, and Mn, consistent with successful bimetallic incorporation. The data revealed high C and N content from organic imidazolate linkers, dominant Zn signals, and trace Mn levels typical for bimetallic ZIF-8 frameworks, with low Mn peak intensities reflecting its minor proportion relative to the Zn matrix (Ismail et al. 2024).

The histogram shows the particle-size distribution curve of Zn-Mn MOF, as shown in Fig. 1d. The average particle size of the MOF ranged from 50 to 100 nm, indicating a narrow, well-controlled size distribution. Such narrow distributions correlate with uniform nucleation and growth conditions and are desired for reproducible adsorption performance (Alnafisah et al. 2025).

The elemental mapping of a Zn-Mn MOF demonstrates the distribution of key elements in the sample. The presence of carbon and nitrogen confirms that the framework contains the imidazolate ligand. Zn mapping verifies the main metal centre of the MOF is present and relatively dispersed. Mn mapping proves manganese is successfully incorporated into the framework, but likely at a lower proportion compared to Zn.

**Fig. 2:**
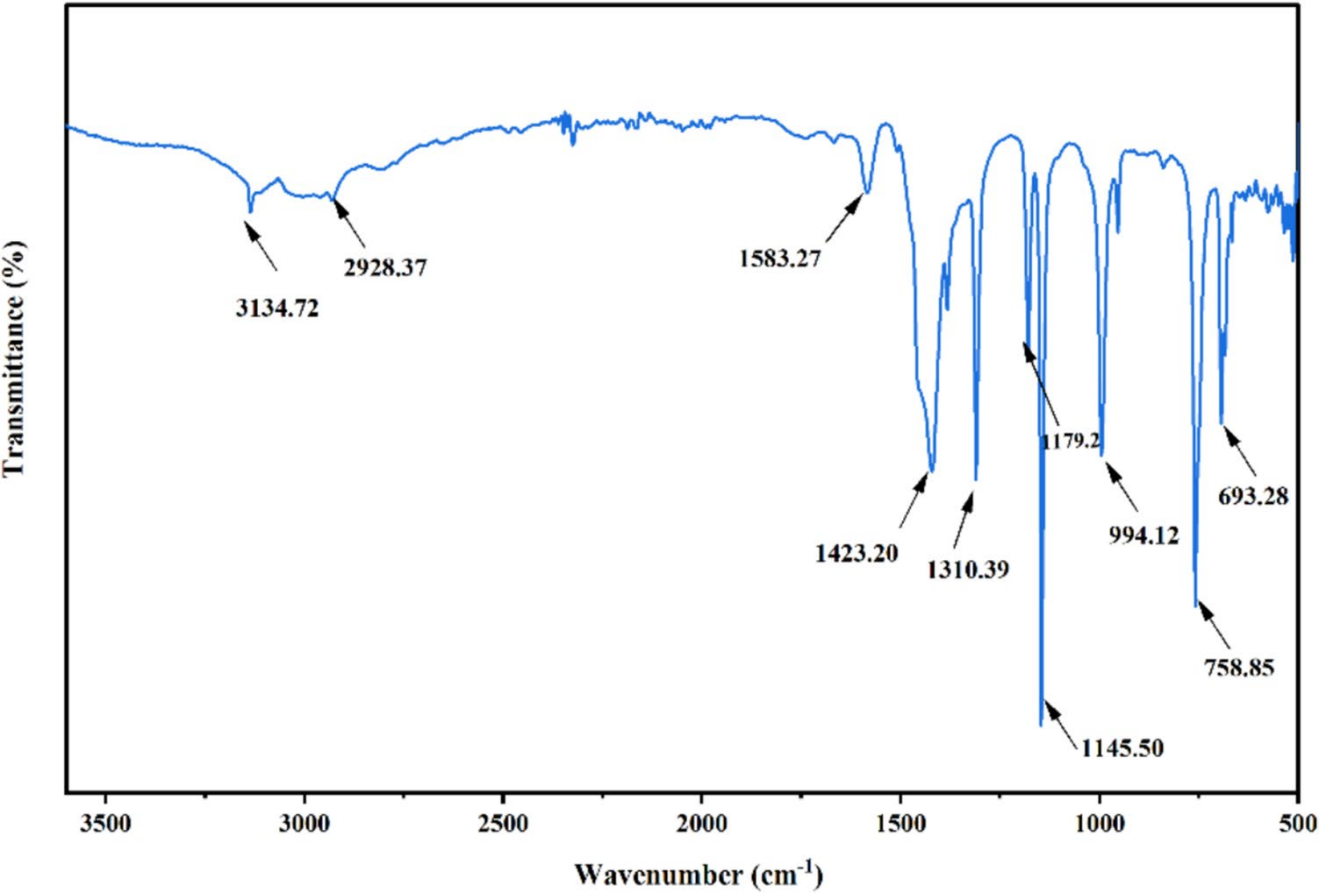
FTIR spectrum of Zn-Mn MOF

The FTIR spectrum of Zn-Mn MOF is demonstrated in Fig. 2. The bands at 3134 and 2928 cm^−1^ correspond to N-H and C-H stretching of the 2-methylimidazole linker, suggesting its incorporation in the framework, while the strong bands at 1583 and 1423 cm^−1^ arise from C=N and C=C stretching vibrations of the imidazole coordinated to Zn and Mn centres (Y. Zhang et al. 2018). The bands in the 1310–1179 and 1145–994 cm^−1^ regions correspond to in-plane C-N and C-H bending, and the features at 758–693 cm^−1^ resemble skeletal vibrations and metal–nitrogen bonds, together verifying a well-formed ZIF-8-type bimetallic structure (Akbarzadeh et al. 2021).

**Fig. 3:**
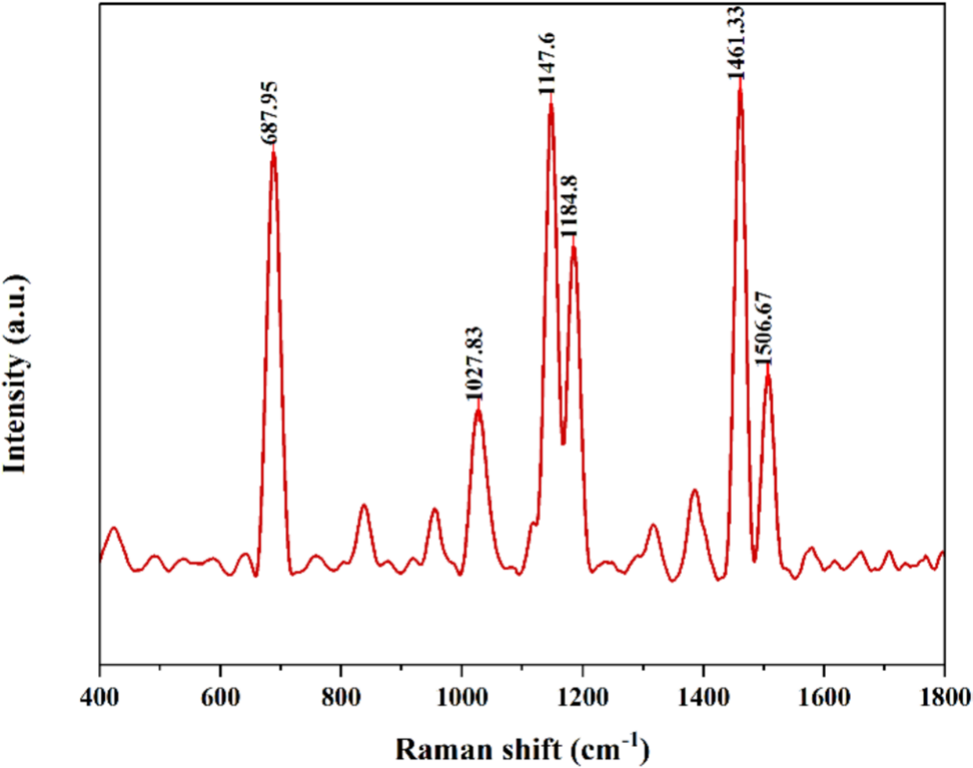
Raman spectrum of Zn-Mn MOF

The Raman spectrum of the Zn-Mn MOF prepared with the 2-methylimidazole linker shows a number of strong peaks that help to identify the structure of the material displayed in Fig. 3. The strong peak at 683.95 cm^−1^ corresponds to stretching modes of the imidazole coordinated to the metal ions. The peaks at 1027.83, 1147.6, and 1184.8 cm^−1^ correspond to C-N stretching, C=N bending, and combined ring modes of the imidazole linker, thus confirming its integrity in the MOF structure (Li et al. 2023). The peaks at 1461.33 and 1506.67 cm^−1^ are attributed to C–H and CH₃ bending modes as well as stretching vibrations within the methyl-substituted imidazole ring. The similarity of these band assignments to previous Raman studies on Zn-based imidazolate frameworks supports the successful synthesis and characteristic bonding motifs in the MOF.

**Fig. 4:**
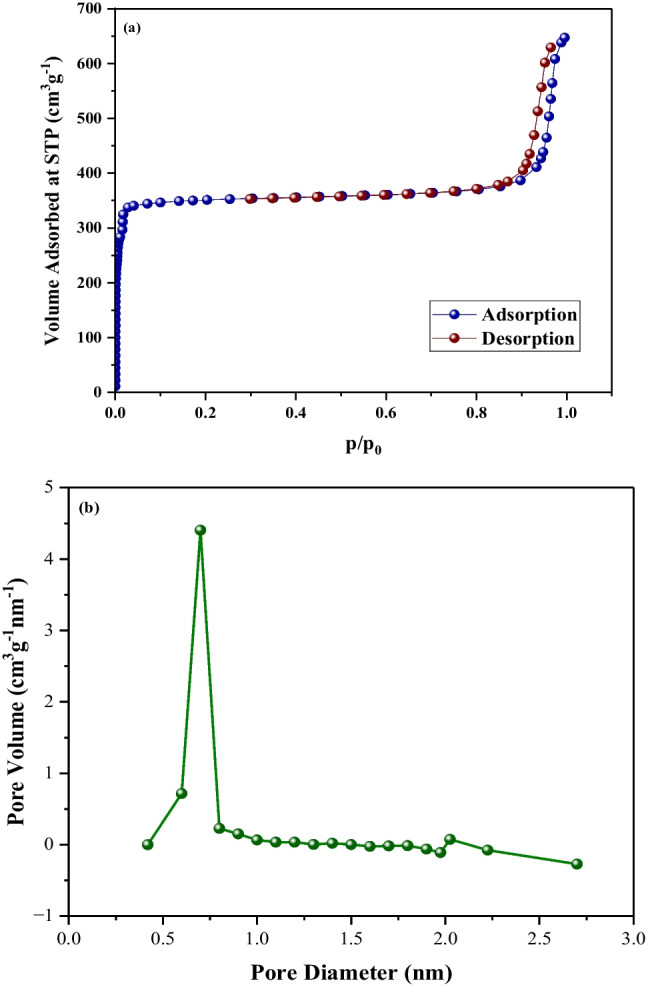
**a** N_2_ adsorption-desorption isotherm and **b** pore size distribution curve for Zn-Mn MOF

Figure 4a shows the N_2_ adsorption–desorption isotherm for the Zn-Mn MOF synthesised with 2-methylimidazole as a linker. The isotherm shows an increase in the uptake of N_2_ at low relative pressures, *p*/*p*₀ < 0.1, typical for type I isotherms according to IUPAC classification and reflects the dominant microporosity of the material’s framework. This isotherm shape is typical for ZIF-8 and its bimetallic derivatives, where the adsorption branch displays a sharp increase due to micropore filling, followed by a plateau and possible slight hysteresis at higher pressures, signalling some contribution from mesopores due to Mn incorporation or framework defects. The surface area for the MOF was found to be 1657.2 m^2^/g, and the pore volume was found to be 0.3625 cm^3^g^−1^. The surface area is significantly higher when compared to Zn-Mn MOFs that used different linkers and solvents. The pore diameter of the material was found to be 0.7 nm, which shows that the material is microporous. The high uptake at low pressure and overall profile confirm a large microporous surface area, crucial for adsorption. Figure 4b represents the Barrett-Joyner-Halenda (BJH) pore diameter distribution curve, which reveals a pronounced peak around 0.9 nm, aligning with the microporous character of the ZIF-8 structure (Ravinayagam and Rehman 2020). The majority of pores fall within the sub-2 nm range, again confirming the dominance of microporosity, but the existence of a small number of larger pores (1–2.5 nm) can originate from interparticle voids or Mn-induced framework modulations. Together, these analyses demonstrate that Zn-Mn MOF retains the classic microporous character of ZIF-8 with some additional mesoporosity, enhancing its effectiveness for selective adsorption.

**Fig. 5:**
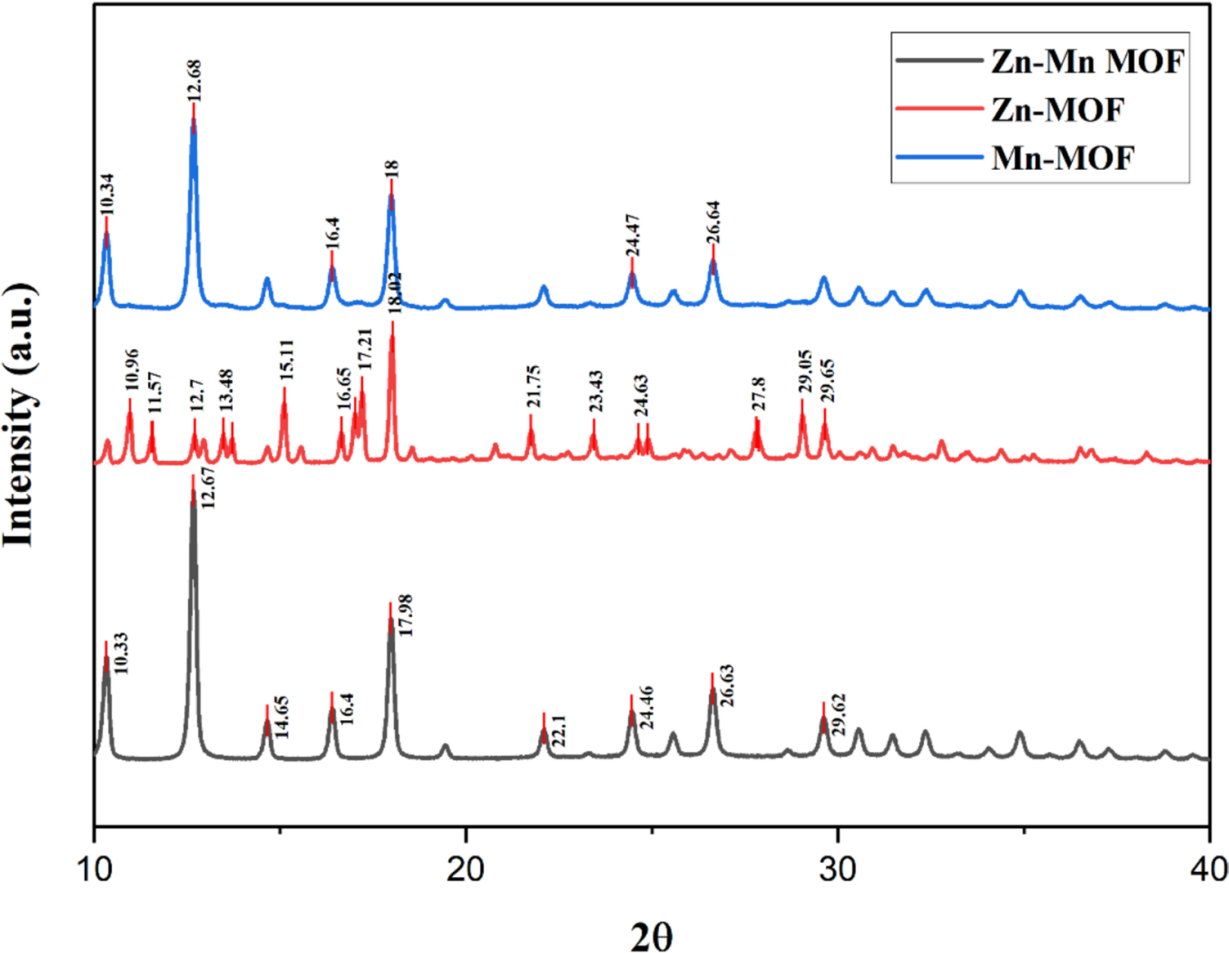
XRD pattern of Mn-MOF, Zn-MOF, and Zn-Mn MOF

The X-ray diffraction (XRD) pattern of the Zn-Mn MOF is illustrated in Fig. 5. It has several sharp peaks at 2*θ* values of 10.4°, 12.7°, 14.7°, 16.4°, 18.0°, and 22.1°, which are characteristic of highly crystalline materials like 2-methylimidazole-derived zeolitic imidazolate frameworks. The peaks appear at nearly the same 2*θ* values with small variations for Zn-MOF and Mn-MOF, signifying that Mn has been successfully incorporated into the Zn-MOF framework. The variations in the positions or intensities of the peaks are due to distortions in the lattice after Mn doping (Abazari et al. 2022; Ullah et al. 2019). Details regarding the d-spacing and crystallite sizes are presented in Table 1.

**Table 1:** Calculated d-spacing and crystallite size of synthesised MOFs

Sample name	d-spacing (Å)	Average crystallite size, *D* (nm)
Zn-MOF	2.72	54.38
Mn-MOF	2.84	3.22
Zn-Mn MOF	2.82	20.13

The diffraction pattern of pristine ZIF-8 aligns closely with the standard reference, verifying phase purity and structural integrity (JCPDS 00–062-1030) (Alowasheeir et al. 2024). Mn-MOF shows a different set of peak modulations, due to the substitution of the central metal from Zn to Mn. Typically, if Mn is doped, XRD peak positions remain similar to those of Zn-MOF but may exhibit slight shifts and broadening, reflecting the ionic radius difference and a mixed metal site occupancy (Howlett et al. 2025).

The average crystallite size of the synthesised MOFs was calculated using Debye-Scherrer equation (Eq. 12) (Fatimah et al. 2022).

12 $$D = 0.89\lambda \beta COS\theta$$

where 0.9 is Scherrer’s constant, *λ* is the X-ray wavelength (1.5406 Å), *β* is the full width at half maximum (FWHM), *D* is the crystallite size, and θ is the Bragg’s angle in radians.

In this study, the average crystallite sizes of the Zn-MOF, Mn-MOF, and Zn-Mn MOF were determined. The calculated d-spacing values for Zn-MOF, Mn-MOF, and Zn-Mn MOF were found to be 2.72, 2.84, and 2.82 Å, respectively. The average crystallite sizes were determined as 54.38 nm for Zn-MOF, 3.22 nm for Mn-MOF, and 20.13 nm for Zn-Mn MOF using the Scherrer equation. The reduction in crystallite size in the bimetallic Zn-Mn MOF compared to pure Zn-MOF indicates that the incorporation of Mn ions influenced crystal growth and structural properties.

**Fig. 6:**
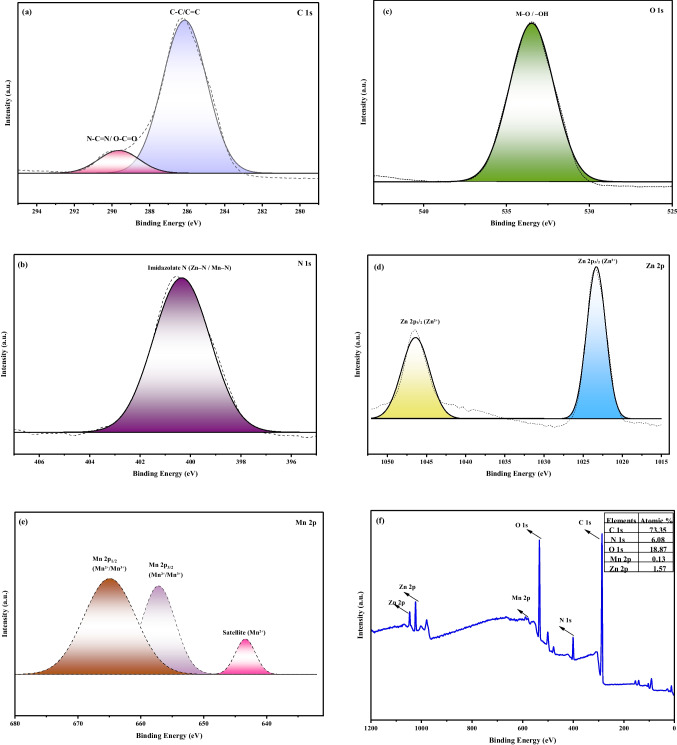
XPS spectra of Zn-Mn MOF. **a** C 1 s, **b** N 1 s, **c** O 1 s, **d** Zn 2p, **e** Mn 2p, and **f** survey

Figure 6 confirms the formation of a Zn-Mn imidazolate framework composed of Zn (II) and mixed-valent Mn centres coordinated by nitrogen-rich organic linkers, yielding a carbon-rich surface typical of 2-methylimidazole-based MOFs. In Fig. 6a, the high-resolution C 1 s spectrum of the Zn-Mn MOF can be deconvoluted into two components. A low-binding-energy peak at 284.7 eV, due to C-C/C=C species from an aromatic imidazole ring and the aliphatic methyl groups of the 2-methylimidazole linker, and a higher binding energy peak at 288.8 eV, attributed to N-C=N units of the imidazolate backbone or surface O-C=O groups (Susi et al. 2015).

From Fig. 6b, the O 1 s spectrum features a dominant peak at 531.8 eV, which can be attributed to metal-oxygen (M-O) bonds (Zn-O/Mn-O) together with surface hydroxyl oxygen. Binding energies in the 531–532 eV region are commonly associated with hydroxyl oxygen and metal carbonate/oxygenated surface groups on the MOF surface (Neophytides et al. 2000).

Figure 6c depicts the high-resolution N 1 s spectrum, in which the predominant peak at 399.6 eV is characteristic of pyridinic/pyrrolic-type nitrogen in N-heterocycles and has been widely assigned to nitrogen in imidazole-like environments. This feature indicates that nitrogen atoms in the 2-methylimidazole linker serve as coordination sites for Zn and Mn, forming M-N bonds within the MOF framework (Artyushkova 2020).

Figure 6d shows that the Zn 2p region consists of two separate peaks at 1022.0 eV and 1045.0 eV, which correspond to Zn 2p₃/₂ and Zn 2p₁/₂ with a spin-orbit splitting characteristic of Zn (II) species. The absence of a satellite peak in the spectrum further confirms the assignment of Zn in the +2 oxidation state and coordinated to nitrogen/oxygen, as typically shown for Zn(II) in ZIF-type and related MOF structures (Santhan and Hwa 2025).

Figure 6e shows the Mn 2p spectrum, which consists of a main peak at 641.9 eV and 653.6 eV, attributed to Mn 2p₃/₂ and Mn 2p₁/₂, respectively. A weak satellite peak is observed at 646.5 eV. These features are consistent with manganese present in mixed Mn^2^⁺/Mn^3^⁺ oxidation states. Such binding energies and satellite structure are commonly reported for Mn-containing MOFs and oxide/carbon composites, indicating partial oxidation of Mn and the coexistence of low and intermediate valent manganese species (Zhao et al. 2018). This significantly enhances Lewis acidity at the metal centre compared to pure Mn^2+^ and Mn^3+^ (d4, Jahn-Teller distorted), which possesses a higher charge density and polarising power than Mn^2+^ (d^5^, high-spin), creating stronger electrophilic sites for coordination with MePr’s nucleophilic oxygen atoms (phenolic -OH, ester C=O).

The XPS survey spectrum reveals contributions from C 1 s, O 1 s, N 1 s, Zn 2p, and Mn 2p as depicted in Fig. 6f. The surface atomic composition of C (73.35%), N (6.08%), O (18.87%), Mn (0.13%), and Zn (1.57%) can be attributed to 2-methylimidazole-based Zn-Mn MOFs or their carbon/oxide derivatives, in which the organic linker dominates the surface. The strong C 1 s and N 1 s signals mainly originate from the organic imidazolate framework, whereas the O 1 s contribution can arise from M-O coordination, surface hydroxyls, adsorbed water, and/or partial oxidation during synthesis or air exposure. The relatively low intensities of Zn 2p and Mn 2p peaks compared with C 1 s and N 1 s are reasonable for a MOF with modest Mn loading and metal nodes partially shielded by an organic-rich surface.

**Fig. 7:**
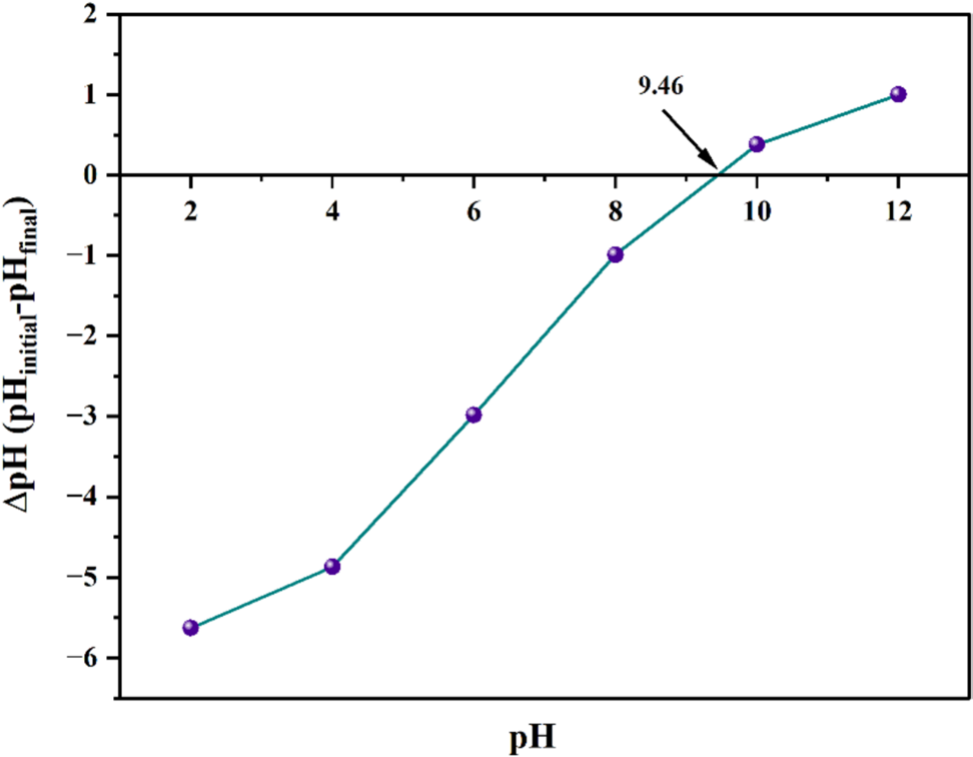
Point zero charge of Zn-Mn MOF

The zero point charge (PZC) is the pH at which the surface charge of the material is neutral. The PZC determines the adsorption behaviour and interaction with ions in solutions. The PZC of the Zn-Mn MOF is given in Fig. 7. The MOF’s surface is positively charged below the PZC and negatively charged above. These characteristics of the MOF, in terms of surface charge, enable it to adsorb certain components selectively. It can attract anions in acidic pH and cations in basic pH (Farooq et al., 2025). The material exhibits a surface point of zero charge (pH_PZC_) of 9.46. Below the pH_PZC_ (pH < 9.46), the MOF surface carries a net positive charge, which improves the adsorption of neutral or weakly negative organic compounds like MePr through non-covalent interactions. However, if Mn is incorporated into Zn-based MOFs derived from 2-methylimidazole, it affects their electronic properties and surface chemistry, which can slightly shift the PZC. Generally, it is evident that Mn in Zn-based MOFs enhances the number of sites on its surface and can change its charge properties by modifying its coordination geometry and electronic structure (Martinez-diaz and Hita 2024).

### Adsorption of MePr with Zn-Mn MOF

#### Effect of pH

**Fig. 8:**
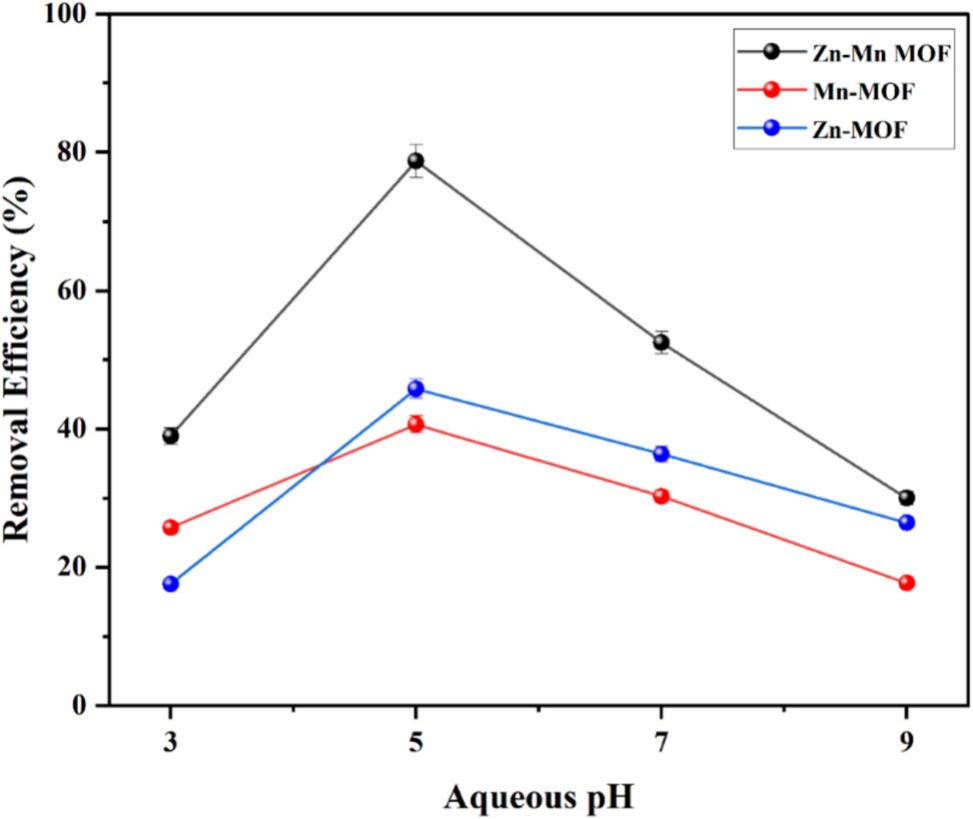
Effect of pH on the MePr adsorption onto Zn-MOF, Mn-MOF, and Zn-Mn MOF (MePr Conc = 10 mg/L; adsorbent dosage = 0.05 g/L; *T* = 25 °C; agitation speed = 150 rpm)

The effect of aqueous phase pH on the adsorption of MePr on Zn-MOF, Mn-MOF, and Zn-Mn MOF was investigated. As illustrated in Fig. 8, all three metal-organic frameworks exhibited a significant dependence on pH, with removal efficiencies increasing from acidic to near-neutral pH and then decreasing under alkaline conditions. At pH 5, the Zn-Mn MOF demonstrated superior adsorption performance, achieving a nearly 80% removal efficiency, which is significantly higher than that of Zn-MOF or Mn-MOF. The synergistic effect from the presence of both metal centres in the framework is attributed to an increased surface area and pore volume, providing more adsorption sites that improve affinity for the MePr binding, and stronger electrostatic and hydrogen-bonding interactions at the optimum conditions (Alaysuy et al. 2024).

Although the PZC of Zn-Mn MOF was determined to be 9.46, the *q*_max_ was observed at pH 5, indicating that optimal adsorption is not governed by surface electrostatics alone. In the pH range of 5–7, the MOF surface carries a positive charge while methyl paraben (MePr, pKa ≈ 8.4) remains predominantly neutral or partially deprotonated, favouring adsorption through non-electrostatic interactions. Specifically, two complementary mechanisms drive MePr uptake: (i) hydrogen bonding between the phenolic –OH and ester groups of MePr and the imidazolate nitrogen/framework oxygen atoms, evidenced by an FTIR (Ellerbrock and Gerke 2021), and (ii) π-π stacking between the aromatic ring of MePr and the 2-methylimidazole linkers through electron donor-acceptor interactions, consistent with computational and experimental studies demonstrating that π-π stacking and hydrogen bonding dominate paraben adsorption on MOFs (González-Hernández et al. 2021). However, at pH 9, the enhanced deprotonation of MePr and the presence of hydroxide ions result in a reduction in the removal efficiency (Daneshgar et al. 2024). Since Zn-Mn MOF exhibited superior adsorption performance compared to its monometallic counterparts, it was selected as the primary adsorbent for further studies.

#### Effect of contact time

**Fig. 9:**
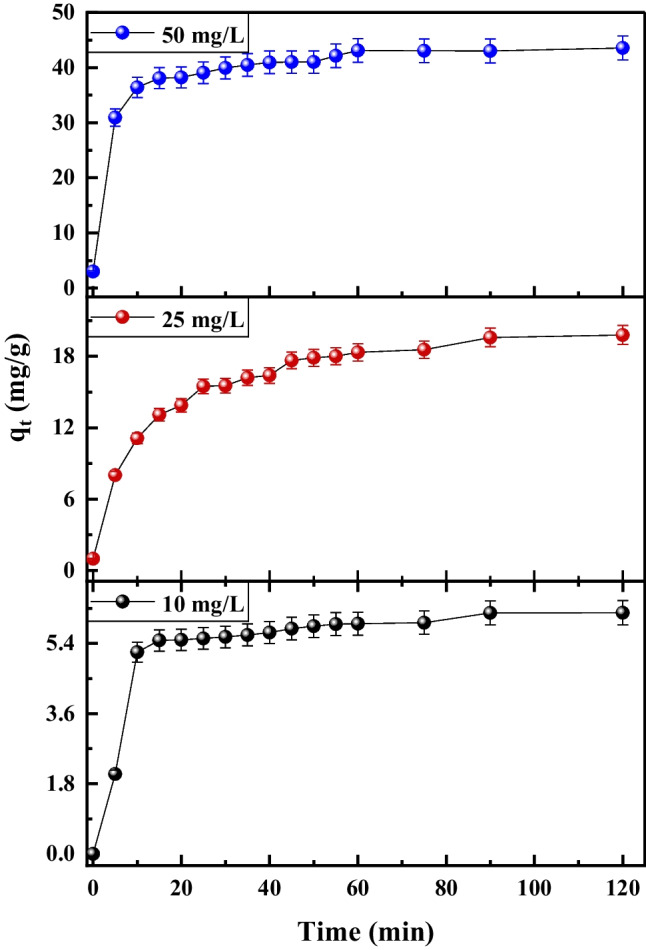
Effect of contact time at varied initial concentrations for the adsorption of MePr onto Zn-Mn MOF (adsorbent dosage = 0.05 g/L; pH = 5; *T* =  25 °C; agitation speed = 150 rpm)

The time-dependent uptake of MePr on the Zn-Mn MOF was investigated at initial concentrations of 10, 25, and 50 mg/L. The amount of MePr adsorbed (*q*_t_) significantly increased during the first 20 min, and then attained equilibrium, which was after 90 min for all three concentrations, as depicted in Fig. 9. The rapid initial rate of adsorption is due to the adsorption sites available on the outer surface, while the second phase is because of intraparticle diffusion and the occupation of inner pores. The higher initial loadings enhanced the equilibrium adsorption capacity (*q*_e_) while maintaining consistent equilibrium time, indicative of efficient pore utilisation independent of concentration. Extending contact time further showed little effect in enhancing adsorption, as most of the active sites were already saturated. Prolonging the adsorption time beyond equilibrium unnecessarily escalates operational and economic costs.

### Adsorption kinetics

**Fig. 10:**
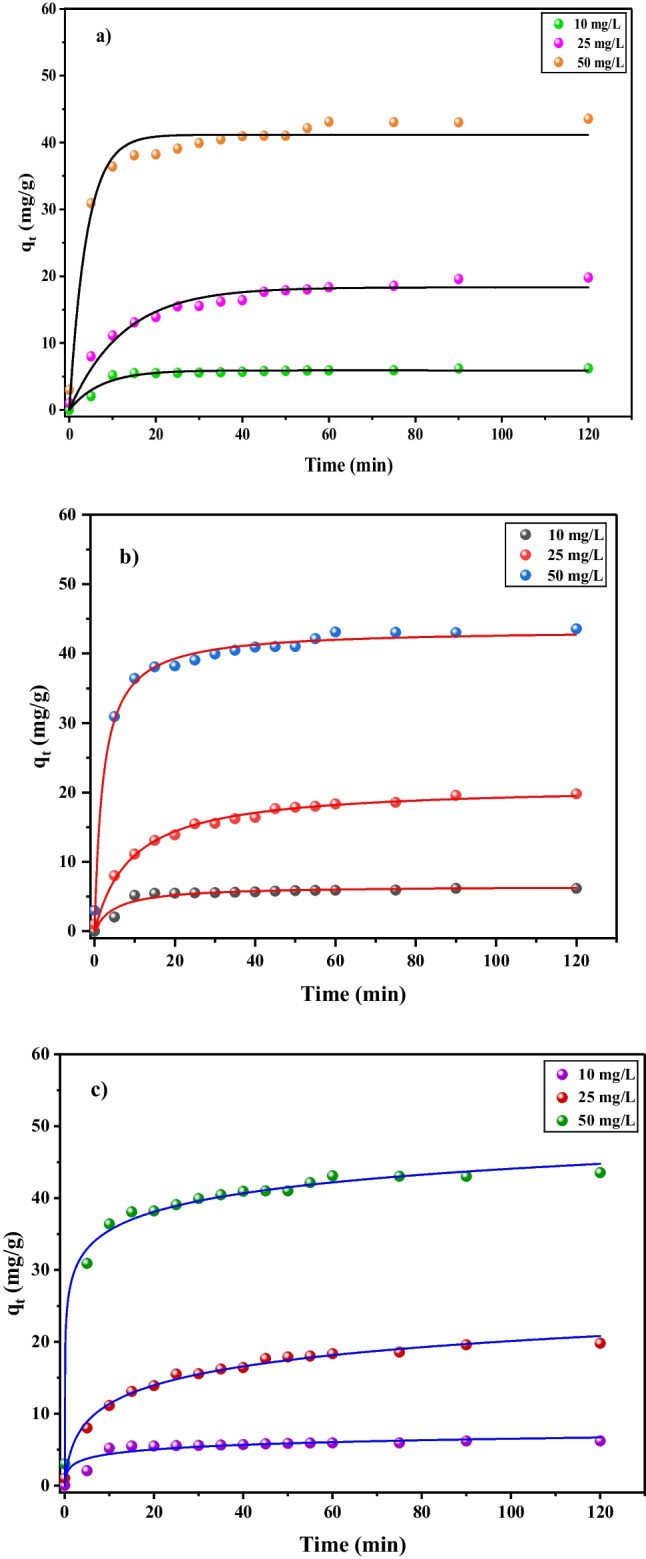
Adsorption kinetics of MePr onto Zn-Mn MOF. **a** Pseudo-first-order kinetic model, **b** pseudo-second-order kinetic model, and **c** Elovich model

The adsorption kinetics of MePr onto Zn-Mn MOF were studied by monitoring the time-dependent uptake at different initial MePr concentrations, as depicted in Fig. 10. An increase in adsorption capacity was observed between 10 and 20 min, followed by a slower approach to equilibrium within about 60–90 min, reflecting the progressive occupation of readily accessible surface sites and subsequent diffusion of MePr into the internal pores. The PFO, PSO, and Elovich equations were used to model the adsorption kinetics. The rate constants, *q*_e_, and *R*^2^ values are tabulated in Table 2. The PSO model described the experimental data best, as verified by higher correlation coefficients, which suggests that the adsorption is driven by chemisorption involving interactions between MePr and the Zn-Mn MOF surface.

**Table 2:** Adsorption kinetic model parameters for methyl paraben adsorption onto Zn-Mn MOF

Kinetic models	Concentration (mg/L)	Parameters	Data
PFO	10 mg/L	*q*_e_ (mg/g) *k*_1_ (min^−1^) *R*^2^ *χ*^2^	5.90 0.13 0.86 0.14
	25 mg/L	*q*_e_ (mg/g) *k*_1_ (min^−1^) *R*^2^ *χ*^2^	18.31 0.08 0.91 1.02
	50 mg/L	*q*_e_ (mg/g) *k*_1_ (min^−1^) *R*^2^ χ^2^	41.07 0.2 0.62 3.23
PSO	10 mg/L	*q*_e_ (mg/g) *R*^2^ *k*_2_ (g/mg min) *χ*^2^	6.52 0.93 0.03 0.02
	25 mg/L	*q*_e_ (mg/g) *R*^2^ *k*_2_ (g/mg min) *χ*^2^	20.99 0.99 0.005 0.15
	50 mg/L	*q*_e_ (mg/g) *R*^2^ *k*_2_ (g/mg min) *χ*^2^	43.49 0.98 0.01 0.87
Elovich model	10 mg/L	*α* (mg/g min) *β* (g/mg) *R*^2^ χ^2^	9.90 1.07 0.87 0.34
	25 mg/L	*α* (mg/g min) *β* (g/mg) *R*^2^ χ^2^	6.81 0.25 0.98 0.30
	50 mg/L	*α* (mg/g min) *β* (g/mg) *R*^2^ *χ*^2^	5.61 0.26 0.98 1.14

### Intraparticle diffusion model

**Fig. 11:**
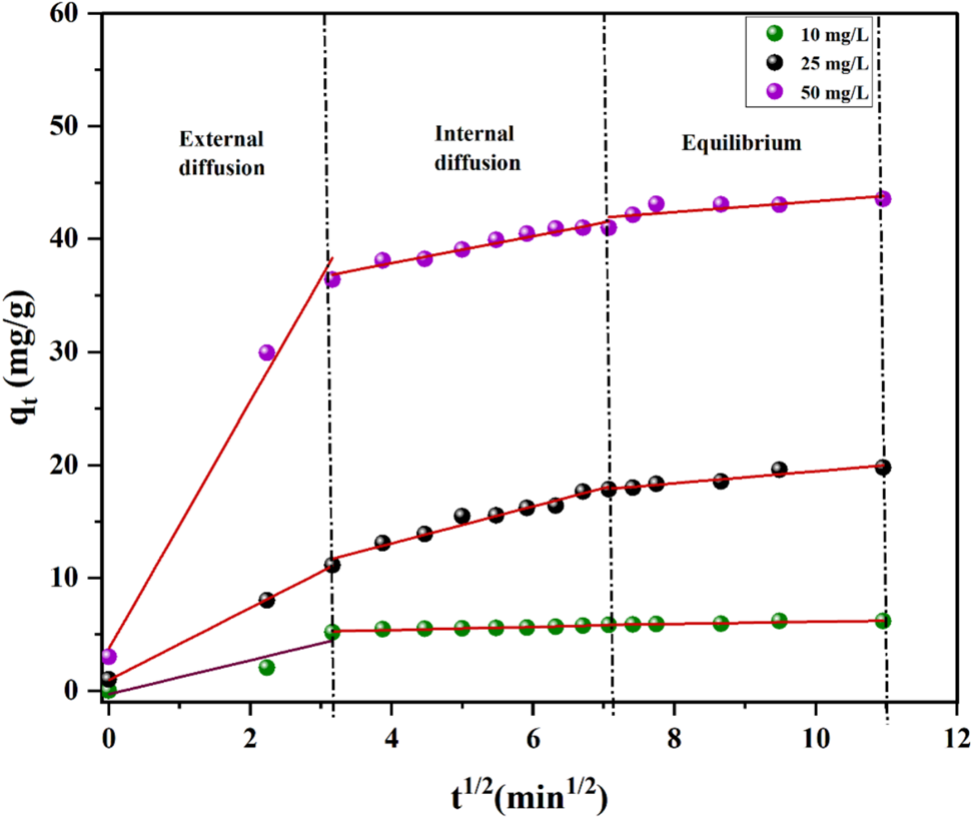
Intraparticle diffusion analysis for MePr adsorption onto Zn-Mn MOF

The intraparticle diffusion model was applied to elucidate the adsorption mechanism of MePr onto Zn-Mn MOF, as depicted in Fig. 11. The corresponding plot was resolved into three linear regions, indicating that the uptake process proceeded through multiple mass-transfer steps rather than a single diffusion-controlled mechanism (De et al. 2013). In the initial stage, the steep slope reflects rapid external diffusion, where adsorption is governed by the abundant availability of vacant active sites and a relatively low boundary-layer resistance. The second region represents internal diffusion, in which the adsorption rate decreases as MePr molecules gradually diffuse into the MOF, and the boundary-layer resistance becomes more pronounced. In the final region, the curve approaches equilibrium, showing the slowest uptake rate as the available adsorption sites become progressively saturated and the system nears equilibrium.

As listed in Table 3, the diffusion rate constants followed the order ${K}_{\mathrm{i}\mathrm{d}(1)}>{K}_{\mathrm{i}\mathrm{d}(2)}>{K}_{\mathrm{i}\mathrm{d}(3)}$ at all initial concentrations, confirming that the adsorption rate was highest in the external diffusion stage and decreased progressively in the subsequent internal diffusion and equilibrium stages. This trend is consistent with concentration-dependent uptake profiles, in which the high initial driving force at 10, 25, and 50 mg/L promoted rapid adsorption at the beginning, followed by slower intraparticle transport and eventual attainment of equilibrium. The nonzero intercepts in each linear segment indicate that intraparticle diffusion was involved but was not the sole rate-limiting step; rather, film diffusion and pore diffusion jointly controlled MePr adsorption onto Zn-Mn MOF (Wang and Guo 2022).

**Table 3:** Intraparticle diffusion model parameters for MePr adsorption on Zn-Mn MOF

Concentration (mg/L)	External diffusion			Internal diffusion			Equilibrium		
	*K*_id(1)_ (mg g^−1^ min^−1/2^)	*C*_1_ (mg g^−1^)	*R*^2^	*K*_id(2)_ (mg g^−1^ min^−1/2^)	*C*_2_ (mg g^−1^)	*R*^2^	*K*_id(3)_ (mg g^−1^ min^−1/2^)	*C*_3_ (mg g^−1^)	*R*^3^
10 mg/L	10.92	3.79	0.98	1.20	33.05	0.95	0.48	38.52	0.58
25 mg/L	3.19	0.97	0.99	1.63	6.52	0.96	0.53	14.15	0.92
50 mg/L	0.39	0.24	0.92	0.14	5.27	0.95	0.09	5.16	0.85

### Effect of adsorbent dosage

**Fig. 12:**
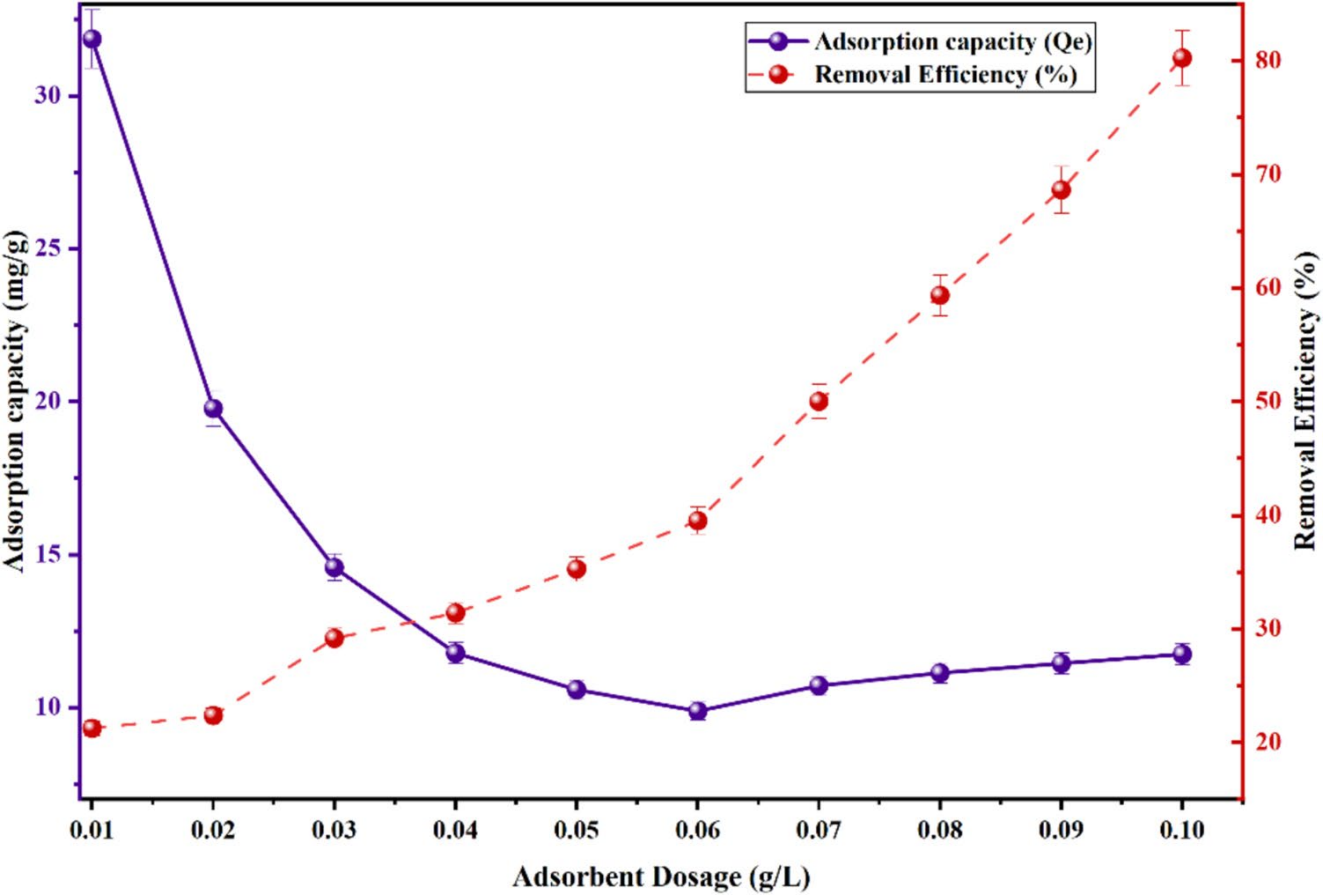
Effect of Zn-Mn MOF dosage on MePr adsorption capacity and removal efficiency. (MePr Conc = 10 mg/L; pH = 5; *T* =  25 °C; agitation speed = 150 rpm)

The influence of the adsorbent dosage on the removal efficiency and adsorption capacity of Zn-Mn MOF towards MePr is shown in Fig. 12. At a lower dosage, the adsorption capacity was about 35 mg/g, but the removal efficiency was about 20% because of the limited surface area available compared to the total amount of MePr in the solution. As the dosage increases, the removal efficiency increases significantly to over 80% at 0.10 g/L, indicating that more active sites are available for the binding of MePr. When the adsorbent dosage was increased, the adsorption capacity reduced and eventually attained equilibrium at higher dosages. This is because of the limited number of active sites and the low adsorbate-to-adsorbent ratio (Elsherbiny et al. 2023).

### Effect of initial concentration of MePr

**Fig. 13:**
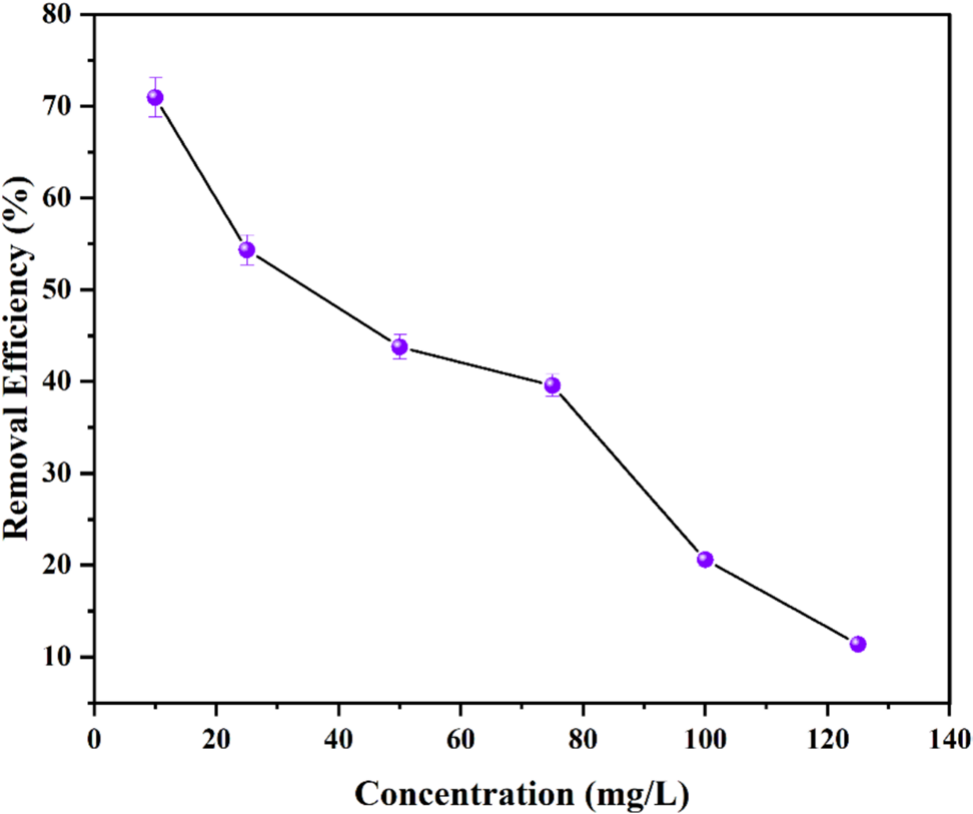
Effect of initial MePr concentration on the removal efficiency of Zn-Mn MOF. (adsorbent dosage = 0.05 g/L; pH = 5; *T* =  25 °C; agitation speed = 150 rpm)

The effect of initial MePr concentration (10–120 mg/L) on the removal efficiency of the Zn-Mn MOF was estimated, as depicted in Fig. 13. At lower concentrations (10 mg/L), Zn-Mn MOF demonstrated a high removal efficiency of approximately 72%, indicating that the available active sites are sufficient to capture most of the adsorbate molecules. However, as the initial concentration of MePr increased, the removal efficiency reduced steadily, reaching 10% at 120 mg/L, which indicated the saturation of the active sites available for adsorption on the fixed adsorbent mass. When MePr concentration is low, the ratio of available adsorption sites to MePr molecules is high, resulting in almost all MePr molecules being captured. As MePr concentration increases, the adsorption sites available for MePr molecules are saturated, thereby lowering the overall removal efficiency (Maghami et al. 2021).

### Adsorption isotherms

**Fig. 14:**
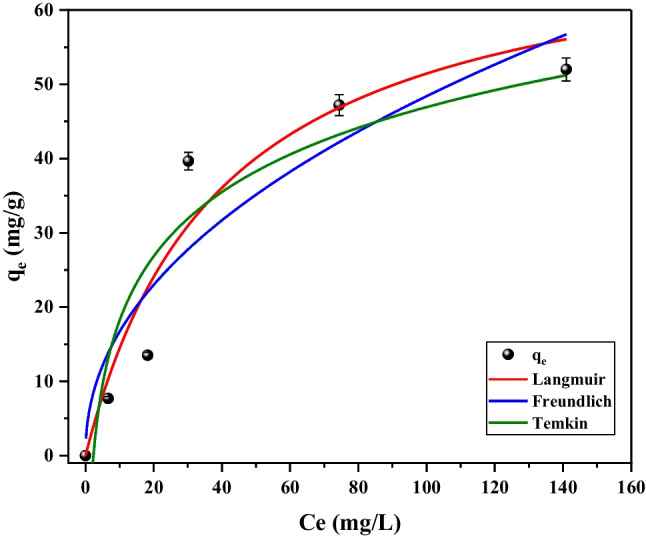
Adsorption isotherm of MePr onto Zn-Mn MOF. Fits of the Langmuir, Freundlich, and Temkin isotherm models

The adsorption isotherm data for methyl paraben on Zn-Mn MOF were explained by the Langmuir, the Freundlich, and the Temkin isotherm models, as depicted in Fig. 14. The Langmuir isotherm provided a monolayer capacity ${q}_{\mathrm{m}}$ of 52.38 mg/g and Langmuir affinity constant ${K}_{\mathrm{L}}$ of 0.062 L mg^−1^. A high correlation coefficient (*R*^2^ = 0.99) and a low chi-square value (*χ*^2^ = 0.5162) indicate that the experimental data are well described by monolayer coverage on a homogeneous surface with a limited number of identical sites, as described in Table 4. This relatively high ${K}_{\mathrm{L}}$ value reflects a strong affinity of methyl paraben molecules for the Zn-Mn MOF surface, in accordance with the steep initial slope of the Langmuir curve. Considering both the determination coefficients and the chi-square statistics of all the isotherm models, the Langmuir isotherm is identified as the appropriate model for the adsorption of MePr onto Zn-Mn MOF. This indicates a predominance of a monolayer adsorption on a nearly homogeneous surface, with Freundlich behaviour capturing secondary heterogeneity effects at lower coverage (Dada et al. 2012). Table 5 summarises the adsorption capacities of various adsorbents used for methyl paraben removal.

**Table 4:** Adsorption isotherm model parameters for MePr adsorption onto Zn-Mn MOF

Isotherm model	Parameters	Data
Langmuir	*q*_m_ (mg/g)	52.38
	*K*_L_ (L/mg)	0.06
	*R*^2^	0.99
	χ^2^	0.51
Freundlich	1/*n*	1.55
	*K*_F_ (L/mg)	8.59
	*R*^2^	0.98
	*χ*^2^	0.10
Temkin	*B*_T_ (J/mol)	16.76
	*A*_T_ (L/mg)	0.21
	*R*^2^	0.88
	*χ*^2^	69.03

**Table 5:** The comparison of the adsorption capacity of various adsorbents for the MePr removal

No	Adsorbent	Surface area (m^2^/g)	Adsorption capacity (mg/g)	References
1	NH_2_-MIL-101(Fe)/Fe_3_O_4_/GO	104.07	13.69	Meng et al. (2024)
2	Waste tamarind fruit shell	-	17.5	Modi et al. (2023)
3	Magnetic cellulose	-	6.2170	Correa-Navarro et al. (2024)
	Microcrystalline cellulose		0.6815	
	Cellulose modified with benzoic acid		1.4625	
4	Magnetic nanoparticles with a phenyl group	6.41	0.6015	Chen et al. (2017)
5	Graphene-NiO composite	-	3.12	Husein (2019)
6	FB-3N (NH_4_Cl modified biochar) FB-3G (milled biochar)	105 6.0	29.21 33.94	Correa-Navarro et al. (2025)
7	AgNPs/nanobiochar/Co-MOF composite	318.3	45.19	Ayinde et al. (2026)
8	Zn-Mn MOF	1657.2	52.38	This study

### Adsorption thermodynamics

**Fig. 15:**
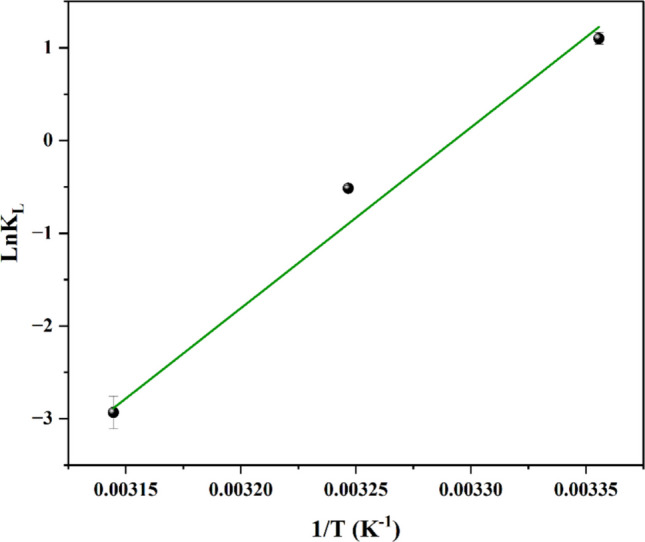
Van’t Hoff’s analysis of MePr adsorption on Zn-Mn MOF

The thermodynamic parameters of MePr adsorption onto Zn-Mn MOF were investigated using batch adsorption experiments conducted at varying temperatures from 293 to 318 K, as illustrated in Fig. 15. The computed thermodynamic parameters (Δ*G*^0^, Δ*S*^0^, and Δ*H*^0^) are given in Table 6. The Langmuir constant (*K*_L_) decreased with temperature from 1.601 L/mg at 298 K to 0.452 L/mg at 308 K and 0.303 L/mg at 318 K, showing a reduction in the affinity of the adsorbate for the MOF surface with increasing temperature. The negative $\Delta {G}^{\circ }$ at 298 K ($-17.17$ kJ mol^−1^) indicates that the adsorption process is thermodynamically spontaneous, but became less negative at 308 K (−16.99 kJ mol^−1^) and 318 K (−16.63 kJ mol^−1^). The negative Δ*H*^0^ (−38.30 kJ mol^−1^) and Δ*S*^0^ (−28.77 J mol^−1^ K^−1^) show that the process is exothermic and accompanied by a decline in entropy, because of the immobilisation of methyl paraben. These parameters can be correlated to the temperature-dependent variation in Δ*G*^0^, which becomes less negative with increasing temperature, signifying that the process becomes thermodynamically less favourable at higher temperatures (Bernal et al. 2021).

**Table 6:** Adsorption thermodynamic parameters for the removal of MePr on Zn-Mn MOF

Adsorbate	Temperature (K)	Langmuir constant (*K*_L_) (L/mg)	Zn-Mn MOF		
			Δ*G*^0^ (kJ mol^−1^)	Δ*H*^0^ (kJ mol^−1^)	Δ*S*^0^ (J mol^−1^ K^−1^)
Methyl paraben	298 K	1.60	−17.16	−38.30	−28.77
	308 K	0.45	−16.99		
	318 K	0.30	−16.62		

### Plausible adsorption mechanism

**Fig. 16:**
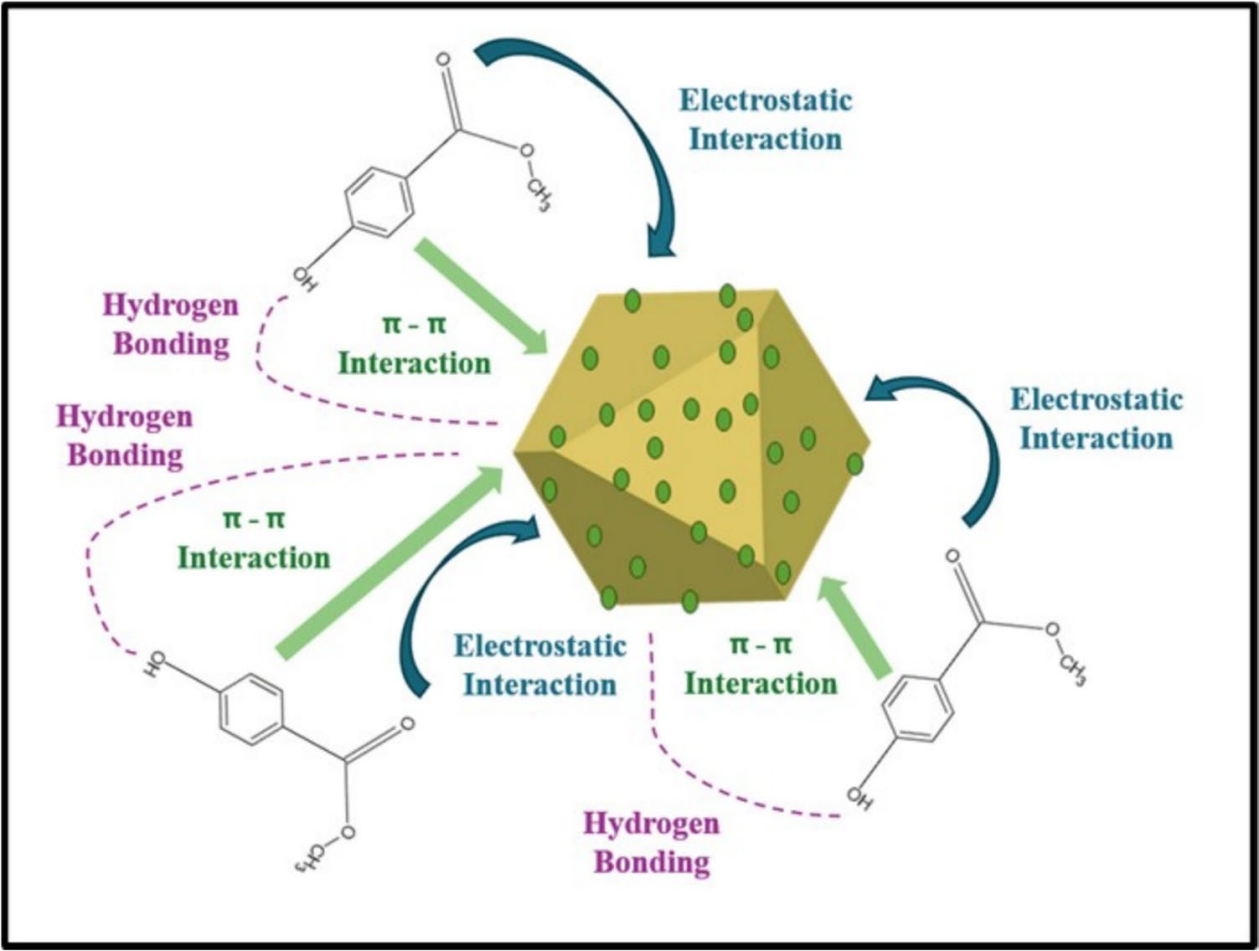
Plausible mechanism for the adsorption of MePr by Zn-Mn MOF

The interaction between the Zn-Mn MOF and MePr is shown in Fig. 16. The adsorption of methyl paraben on Zn-Mn MOF is a complex process involving simultaneous interactions and mechanisms. As shown in Fig. 15, the main driving forces for the adsorption are hydrogen bonding and π-π stacking interactions. The hydroxyl and carbonyl functional groups of methyl paraben can form hydrogen bonds with the surface -OH or -NH groups of the Zn-Mn MOF, while the aromatic ring of methyl paraben can interact with the π-electron-rich aromatic rings or imidazole of the MOF through π-π stacking interactions. These interactions can help to create strong molecular affinity and stable adsorption on the MOF surface. Based on the kinetic studies, the PSO model was the best fit, further supporting that chemisorption is the rate-controlling step, which involves specific interactions between the adsorbent and MePr. The equilibrium data were best fitted by the Langmuir isotherm, indicating predominant monolayer adsorption of MePr onto a finite number of energetically uniform sites with strong specific MePr–surface interactions. A negative enthalpy change signifies an exothermic adsorption process, in which the uptake of MePr releases heat and is energetically favourable. Overall, the adsorption of MePr on Zn-Mn MOF is governed by hydrogen bonding and π-π interactions, fitting PSO kinetics and the Langmuir isotherm, and proceeds as an exothermic chemisorption-driven process.

### Reusability study

**Fig. 17:**
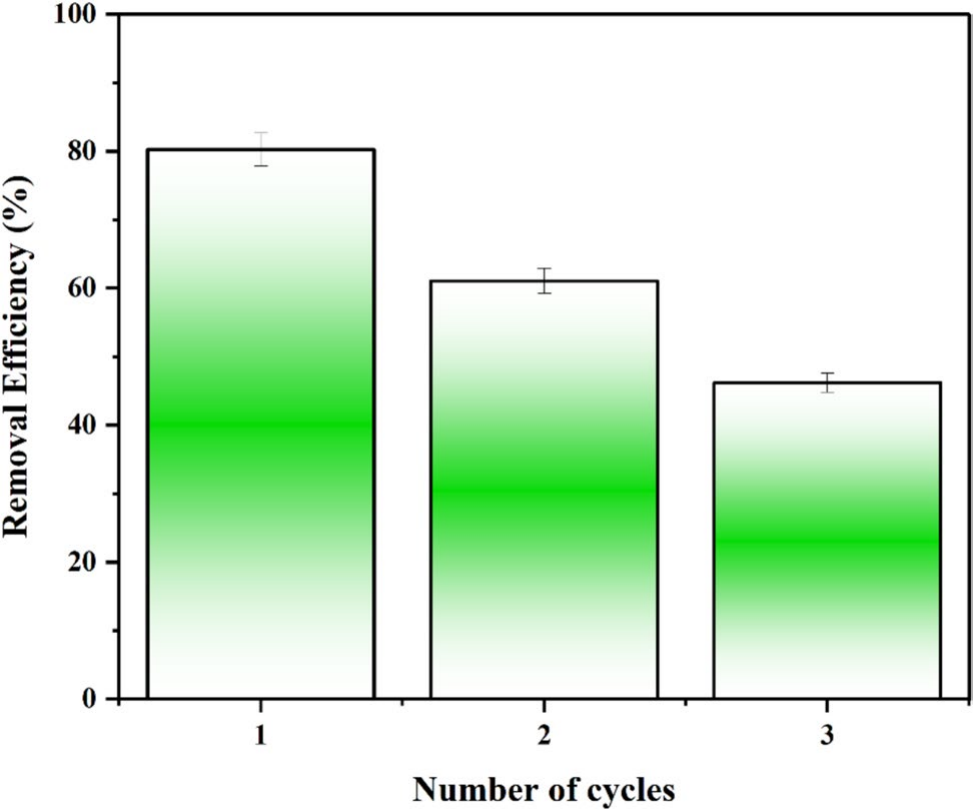
Reusability of Zn-Mn MOF for the removal of MePr

The reusability of the adsorbent is also essential for environmental applications. The recyclability of the Zn-Mn MOF was evaluated for MePr adsorption under the optimised conditions. After each cycle, the used MOF was separated, washed with ethanol to remove MePr, and then dried for reuse under the same conditions. As shown in Fig. 17, removal efficiency decreases from the first to the second cycle, followed by a gradual decline in subsequent cycles.

This trend indicates that some high-energy adsorption sites are strongly occupied after the first cycle, while the remaining sites remain active across multiple cycles. The gradual loss in capacity is consistent with a chemisorption-dominated mechanism defined by the PSO kinetic model and Langmuir monolayer adsorption, where strong hydrogen bonding/π-π interactions and possible coordination to metal centres reduce the number of fully regenerable active sites upon repeated use (Figuerola et al. 2025).

### Methyl paraben-spiked water matrices

**Fig. 18:**
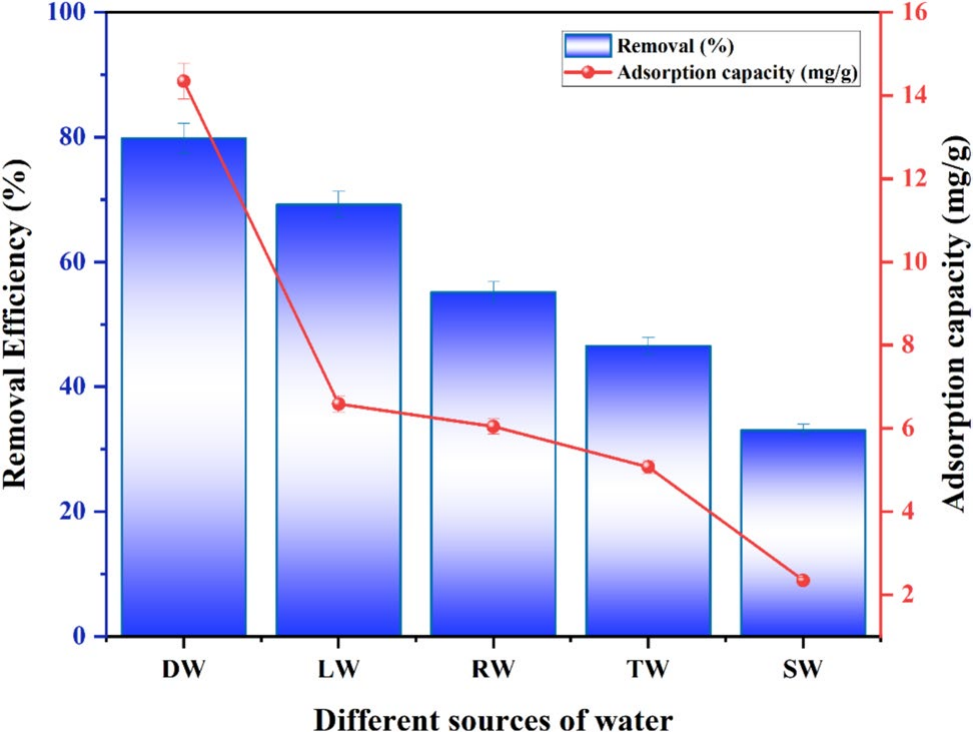
Zn-Mn MOF for the removal of MePr spiked in different water matrices

MePr spiked in water matrix experiments were conducted by spiking a MePr solution into different real water samples (distilled, lake, river, tap, and seawater) and performing adsorption with Zn-Mn MOF under optimised conditions (initial MePr concentration, contact time, and adsorbent dosage). In distilled water (DW), Zn-Mn MOF exhibited higher performance, achieving 80% removal and an adsorption capacity of 14 mg/g, as illustrated in Fig. 18. In contrast, the removal efficiencies and capacities decreased in more mineralised matrices: 70% and 12 mg/g in lake water (LW), 50% and 9 mg/g in river water (RW), 40% and 8 mg/g in tap water (TW), and 30% and 6 mg/g in seawater (SW), respectively. The monotonic decline from DW to SW is attributed to matrix interference effects arising from coexisting ions and dissolved organic matter. Background cations such as Ca^2+^, Mg^2+^, Na^+^, and other dissolved solids compete with MePr molecules for the active adsorption sites of Zn-Mn MOF, thereby weakening electrostatic and hydrogen-bonding interactions. Furthermore, natural organic matter present in natural water samples can accumulate on the adsorbent surface and partially block pore channels, thereby reducing the accessibility of active sites and hindering MePr diffusion. The high ionic strength and salinity of seawater further suppress adsorption through diffusion limitations and competitive interactions, resulting in the lowest removal efficiency (Kuo et al. 2019). Despite this matrix effect, Zn-Mn MOF still exhibits considerable MePr uptake across all tested water samples, displaying its potential for real-world water treatment applications.

**Fig. 19:**
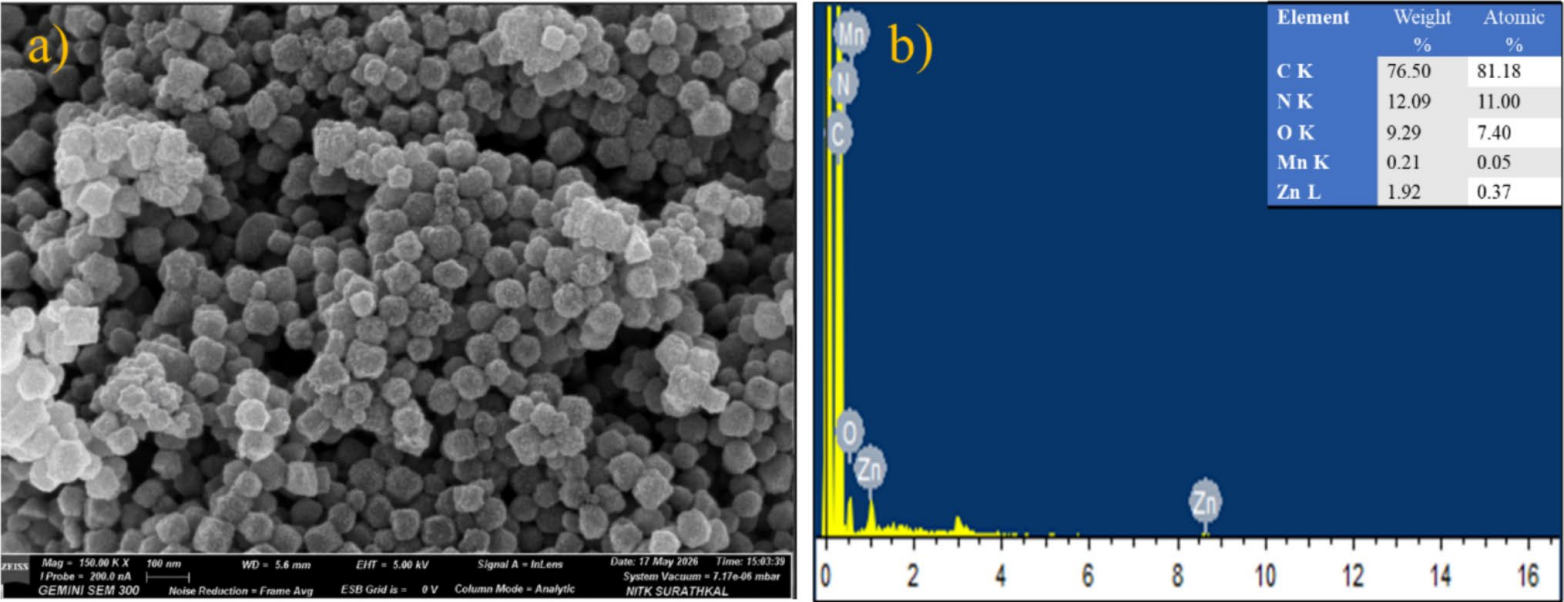
FE-SEM images of Zn-Mn MOF after adsorption of MePr

After adsorption (Fig. 19), the FE-SEM image showed noticeable surface modifications and increased agglomeration of the particles, which may be attributed to the interaction and adsorption of MePr molecules onto the surface of the Zn-Mn MOF. The changes in elemental weight percentages were observed, particularly an increase in carbon content, suggesting the successful adsorption of MePr molecules onto the MOF surface. An additional oxygen (O) peak appeared in the EDAX spectrum along with changes in elemental composition, confirming the successful adsorption of MePr molecules onto the Zn-Mn MOF surface. The presence of oxygen may be attributed to the oxygen-containing functional groups of MePr. Moreover, the Zn and Mn peaks remained visible after adsorption, indicating that the structural stability of the Zn-Mn MOF was maintained throughout the adsorption process.

**Fig. 20:**
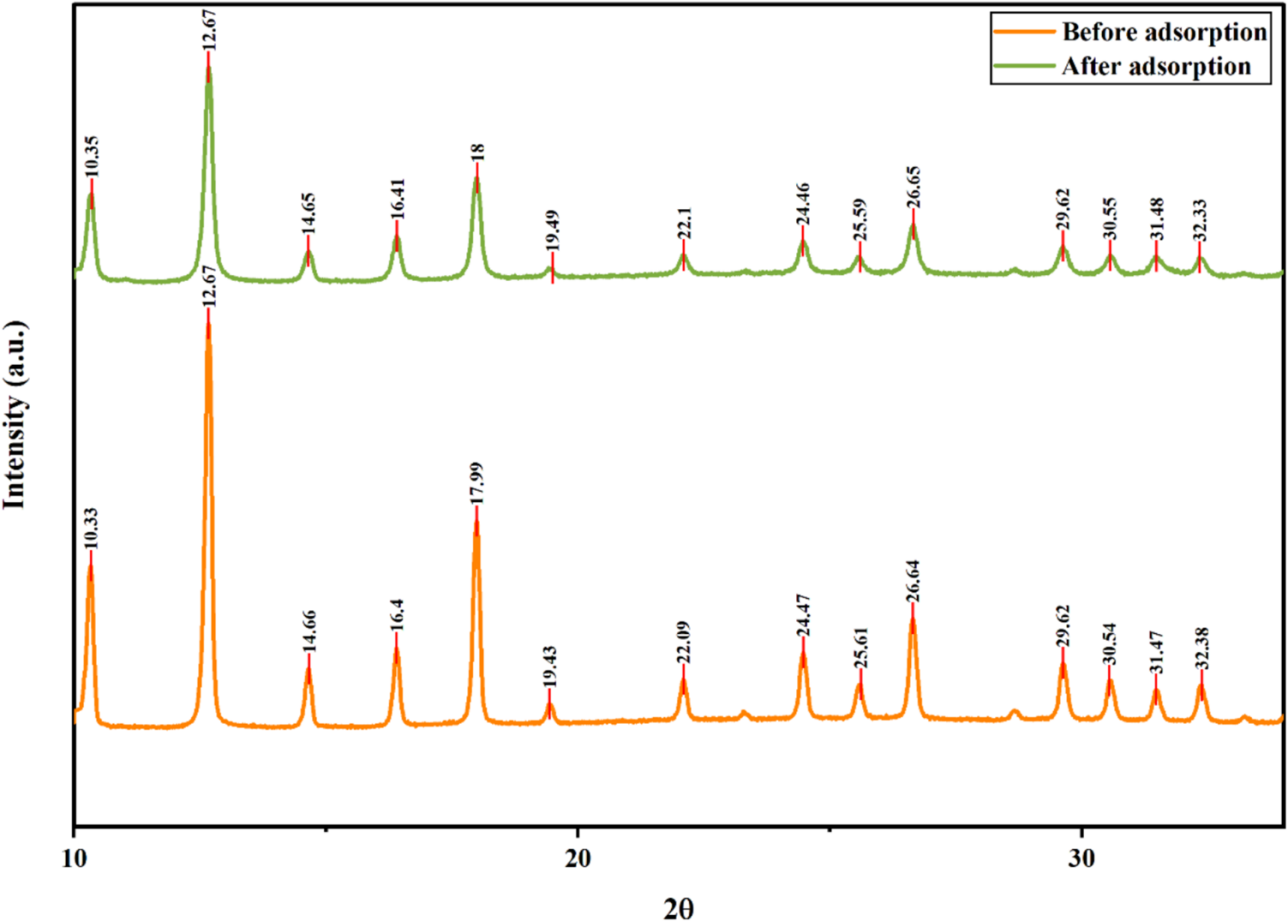
XRD pattern of Zn-Mn MOF after adsorption of MePr

After adsorption of MePr (Fig. 20), the XRD pattern retained all the characteristic diffraction peaks at similar 2θ positions, with slight peak shifting and marginal variation in intensity. The major reflections appeared at 10.35°, 12.67°, 14.65°, 16.41°, 18.00°, 19.49°, 22.10°, 24.46°, 25.59°, 26.65°, 29.62°, 30.55°, 31.48°, and 32.33°. The preservation of the characteristic peaks after adsorption indicates that the crystalline framework of the Zn-Mn MOF remained structurally stable during the adsorption process.

A slight reduction in peak intensity along with minor shifts may be attributed to the interaction of methyl paraben molecules with the active sites of the MOF structure. These changes suggest successful adsorption of MePr onto the Zn-Mn MOF surface without causing significant collapse or destruction of the framework. The absence of new impurity peaks after adsorption further confirms that no secondary crystalline phases were formed during the adsorption process.

**Fig. 21:**
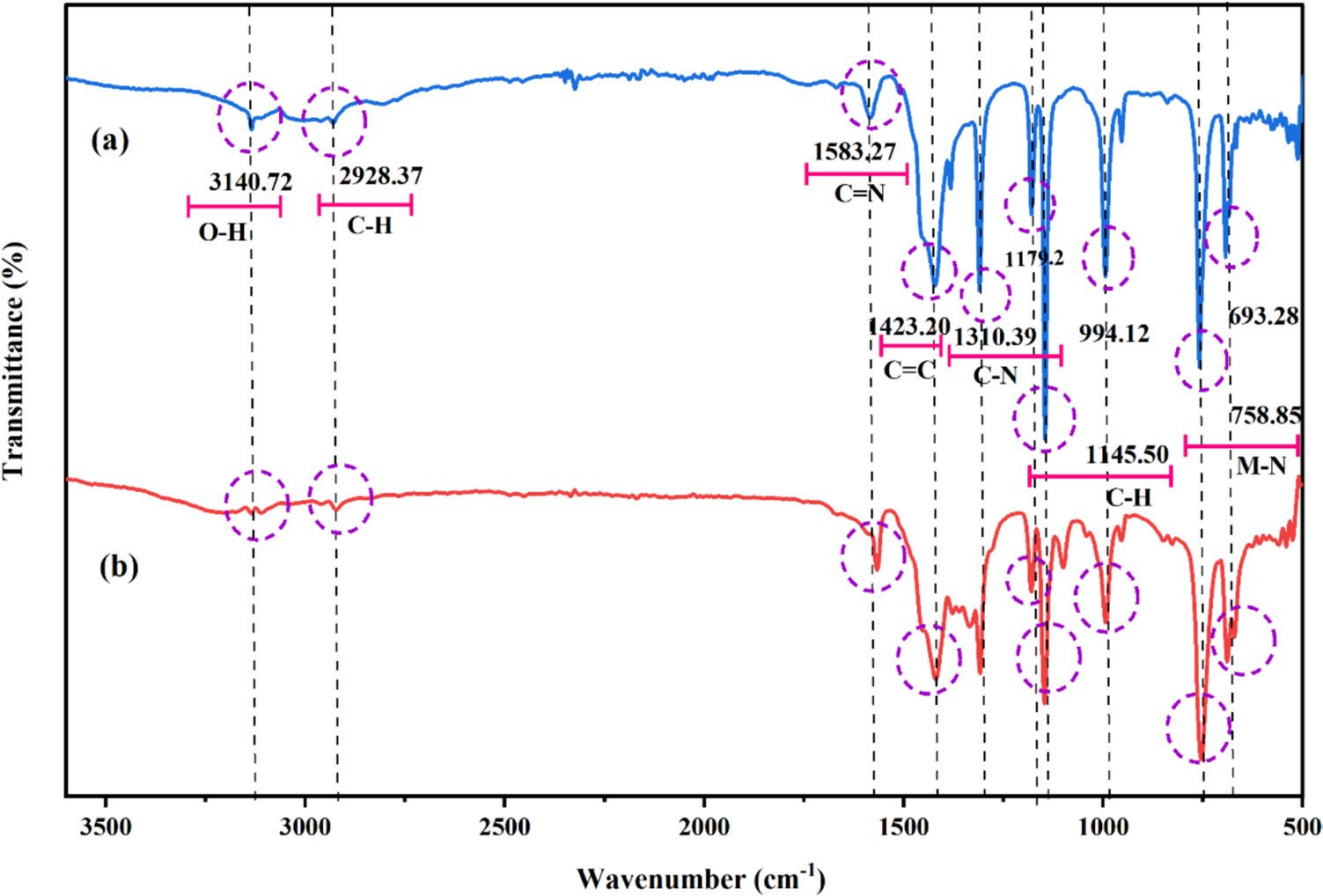
FTIR spectra of Zn-Mn MOF before (**a**) and after (**b**) adsorption of MePr

After adsorption of MePr (spectrum b), the main framework bands of Zn-Mn MOF are preserved, indicating that the MOF structure remains intact, but noticeable intensity changes and slight shifts are observed in key regions, as illustrated in Fig. 21. The broad O-H/C-H stretching band at 3140–2928 cm^−1^ becomes slightly broader, while the C=N/C=C region (1583–1423 cm^−1^) exhibits small changes in position, which can be assigned to hydrogen bonding interactions between the phenolic -OH and ester C=O of MePr and the imidazolate N atoms or surface -OH groups of the metal nodes. The persistence of the metal-N-N skeletal absorptions at 758–693 cm^−1^ in the loaded sample indicates that there is no framework degradation during the adsorption process, and thus, the observed spectral changes can be assigned to MePr-MOF interactions.

## Conclusions

In this study, a manganese-doped ZIF-8 metal-organic framework (Zn-Mn MOF) was synthesised at room temperature. This advanced material exhibited an exceptional surface area and good adsorption capacity for the target contaminant, methyl paraben. The characterisation of field emission scanning electron microscopy confirmed the polyhedral shape, thus proving the uniform particle formation characteristic of ZIF-type frameworks. Further spectroscopic analysis using FTIR and Raman spectroscopy confirmed the imidazolate-based MOF structure formation through characteristic vibrational bands, thus ensuring the successful integration of 2-methylimidazole ligands. Nitrogen adsorption-desorption isotherms confirmed that the adsorption type was of type-1, which was characteristic of high porosity and surface area, essential for efficient adsorbent functionality. The XPS results confirm the framework with Zn (II) and Mn2⁺/Mn3⁺ ions coordinated with 2-methylimidazole ligands, which have a high carbon content on the surface (C 73.35%, N 6.08%). Adsorption studies identified optimal conditions achieving a maximum removal efficiency of 80%. The adsorption kinetics followed a pseudo-second-order model, suggesting chemisorption. The isotherm study fitted well to the Langmuir model. Reusability studies confirmed the potential of Zn-Mn MOF, as ethanol successfully regenerated the adsorbent for three consecutive cycles. In conclusion, Zn-Mn MOF is a highly efficient, cost-effective adsorbent for removing methyl paraben from aqueous solutions, with significant potential for water remediation.

## Data Availability

Data will be available on request.
